# *LncRNA-HIT* Functions as an Epigenetic Regulator of Chondrogenesis through Its Recruitment of p100/CBP Complexes

**DOI:** 10.1371/journal.pgen.1005680

**Published:** 2015-12-03

**Authors:** Hanqian L. Carlson, Jeffrey J. Quinn, Yul W. Yang, Chelsea K. Thornburg, Howard Y. Chang, H. Scott Stadler

**Affiliations:** 1 Skeletal Biology Program, Shriners Hospitals for Children, Portland, Oregon, United States of America; 2 Program in Epithelial Biology, Howard Hughes Medical Institute, Stanford University School of Medicine, Stanford, California, United States of America; 3 Department of Biochemistry & Molecular Biology, Michigan State University, East Lansing, Michigan, United States of America; 4 Department of Molecular and Medical Genetics, Oregon Health & Science University, Portland, Oregon, United States of America; Murdoch Childrens Research Institute, AUSTRALIA

## Abstract

Gene expression profiling in E 11 mouse embryos identified high expression of the long noncoding RNA (lncRNA), *LNCRNA-HIT* in the undifferentiated limb mesenchyme, gut, and developing genital tubercle. In the limb mesenchyme, *LncRNA-HIT* was found to be retained in the nucleus, forming a complex with p100 and CBP. Analysis of the genome-wide distribution of *LncRNA-HIT*-p100/CBP complexes by ChIRP-seq revealed *LncRNA-HIT* associated peaks at multiple loci in the murine genome. Ontological analysis of the genes contacted by *LncRNA-HIT-*p100/CBP complexes indicate a primary role for these loci in chondrogenic differentiation. Functional analysis using siRNA-mediated reductions in *LncRNA-HIT* or p100 transcripts revealed a significant decrease in expression of many of the *LncRNA-HIT*-associated loci. *LncRNA-HIT* siRNA treatments also impacted the ability of the limb mesenchyme to form cartilage, reducing mesenchymal cell condensation and the formation of cartilage nodules. Mechanistically the *LncRNA-HIT* siRNA treatments impacted pro-chondrogenic gene expression by reducing H3K27ac or p100 activity, confirming that *LncRNA-HIT* is essential for chondrogenic differentiation in the limb mesenchyme. Taken together, these findings reveal a fundamental epigenetic mechanism functioning during early limb development, using *LncRNA-HIT* and its associated proteins to promote the expression of multiple genes whose products are necessary for the formation of cartilage.

## Author Summary

A fundamental problem studied by skeletal biologists is the development of regenerative therapies to replace cartilage tissues impacted by injury or disease, which for individuals affected by osteoarthritis represents nearly half of all of all adults over the age of sixty five. To date, no therapies exist to promote sustained cartilage regeneration, as we have not been able to recapitulate the programming events necessary to instruct cells to form articular cartilage without these cells continuing to differentiate into bone. Our analysis of the early programming events occurring during cartilage formation led to the identification of *LncRNA-HIT* a long noncoding RNA that is essential for the differentiation of the embryonic limb mesenchyme into cartilage. A genome wide analysis of *LncRNA-HIT’s* distribution in the mesenchyme revealed strong association between *LncRNA-HIT* and numerous genes whose products facilitate cartilage formation. In the absence of *LncRNA-HIT*, the expression of these chondrogenic genes is severely reduced, impacting the differentiation of these cells into cartilage. Mechanistically, *LncRNA-HIT* regulates these pro-chondrogenic genes by recruiting p100 and CBP to these loci, facilitating H3K27ac and transcriptional activation. *LncRNA-HIT* also appears to be present in most vertebrate species, suggesting that the epigenetic program regulated by this lncRNA may represent a fundamental mechanism used by many species to promote cartilage formation.

## Introduction

In the animal kingdom, embryogenesis proceeds through the coordinated expression of genes whose products mediate the formation of complex tissues and structures. While proteins encoded by mRNAs contribute extensively to the regulation of these developmental processes, recent studies of the human and mouse genomes suggest that long noncoding RNAs (lncRNAs) play an essential role in coordinating the expression of genes required for tissue formation and organ development [1–5].

As gene regulatory molecules, lncRNAs modulate target gene expression using a variety of mechanisms, particularly in the nucleus where they can function as decoys, scaffolds, guides, or even enhancers [6]. As decoys, lncRNAs can titrate away transcription factors (*GAS5*-Glucocorticoid receptor), the transcriptional machinery (*DHFR minor*-TFIIB) or even splicing factors (*MALAT1*- SR splicing factors) to modulate the expression of target genes [7–9]. As a component of their secondary structures, lncRNAs such as *HOTAIR*, *ANRIL*, and *Kcnq1ot1* provide protein-specific scaffolds, assembling enzyme complexes such as the polycomb repressive complexes 1 or 2 (PRC1, PRC2), LSD-1, and CoREST-HDAC to facilitate changes in histone lysine methylation or acetylation to enforce the transcriptional state of a specific locus [10–14]. Nuclear lncRNAs may also function as guides to localize protein complexes to specific chromosomal regions. Notably, the lncRNA, *XIST*, functions as a guide to recruit proteins such as YY1 or components of PRC2 to promote X chromosome inactivation [15–19]. Finally a growing body of evidence indicates that lncRNAs may also function as enhancer RNAs, using chromosomal looping to place proteins bound by the lncRNA proximal to genes to facilitate their regulation [5, 20–22].

A key group of genes regulated by lncRNAs are the Hox genes, a conserved family of developmental transcription factors that exhibit temporally- and spatially-restricted domains of expression and function [5, 23–29]. Evidence for the unique functions of the vertebrate *Hox* lncRNAs was first shown with the *trans*-acting functions of *HOTAIR*, which is expressed from the HoxC locus to recruit PRC2 to the HOXD cluster, where it mediates H3K27 methylation to repress the expression of several 5’ HoxD genes [4]. During development, the 5’ HoxD proteins regulate limb and axial skeleton development, suggesting that de-repression of the 5’ HoxD genes, by perturbations in *HOTAIR* expression, would most likely affect these same skeletal elements [30–32]. Recent studies confirm this hypothesis, as mice lacking *HOTAIR* exhibit malformations of carpal/metacarpal elements in the limb as well as homeotic transformations of the lumbar, sacral, and caudal vertebrae, phenotypes consistent with de-repression of *Hoxd11* and *Hoxd13* [29].

Proximal to the 5’ HoxA gene cluster is the lncRNA *HOTTIP*, which functions as an enhancer lncRNA to regulate the expression of 5’ HoxA genes to control the growth and elongation of zeugopod and autopod skeletal elements [5]. Mechanistically, *HOTTIP* modulates gene expression by chromosomal looping, placing its recruited Trithorax/WDR5/MLL protein complexes proximal to 5’ HoxA genes to facilitate H3K4me3 and gene expression [5,33]. *HOTTIP* function has also been associated with endochondral ossification [34]. This finding in conjunction with the functional studies of *HOTAIR* indicate a role for lncRNAs associated with the vertebrate Hox genes as necessary components for the development, patterning, and maturation of skeletal tissues [5,29,34]. Interestingly, a second lncRNA, *LncRNA-HIT*, has also been identified within the HOXA locus [35]. While initially characterized as a TGFβ-induced modulator of epithelial-mesenchymal transformations in tumor cells [35] our subsequent analysis of this lncRNA indicates that it is also expressed in the early limb where we hypothesized a role for this lncRNA in mediating chondrogenic differentiation. Characterization of subcellular localization of *LncRNA-HIT* by RNA FISH indicated the transcript is predominantly localized to the nucleus where it associates with p100/CBP complexes, suggesting that *LncRNA-HIT* may regulate gene expression by recruiting modulators of H3K27 acetylation. To identify the genes most likely to be regulated by *LncRNA-HIT*, ChIRP-seq was performed using pre-chondrogenic limb bud tissue. From this analysis *LncRNA-HIT* was found to be associated within 25 kb of numerous pro-chondrogenic genes including: *Bmpr1b*, *Gli2*, *Col14a1*, *Adam17*, *Kdelr2*, *Pik3cb*, *Hoxa13*, *Hoxa11*, *Ncam1*, and *Gpc6*. Loss of function analyses confirmed *LncRNA-HIT ‘s* role as a modulator of pro-chondrogenic expression as, H3K27ac, and near peak gene expression were significantly reduced by *LncRNA-HIT*-specific siRNA treatments in the limb mesenchyme. Moreover, chondrogenic differentiation was also significantly reduced in limb mesenchyme treated with siRNAs targeting either *LncRNA-HIT* or the p100 transcript, confirming that the *LncRNA-HIT* and its recruited protein complex are necessary to maintain H3K27ac and chondrogenic gene expression which facilitates differentiation. Combined, these findings identify an epigenetic mechanism functioning during limb skeletogenesis, using an lncRNA to coordinate the expression genes necessary to direct undifferentiated limb mesenchyme towards a chondrogenic state.

## Results

### Developmental expression of *LncRNA-HIT* reveals both distinct and similar domains of expression with 5’ *HOX* genes

The *LncRNA-HIT* transcript was first identified as the full length cDNA, *9530018H14RIK*, by the RIKEN Mouse Gene Encyclopedia Project and was mapped as a single exon to mouse chromosome 6 between *Hoxa11* and *Hoxa13* by genome sequencing (S1 Fig) [36]. Conservation analysis of the *LncRNA-HIT* cDNA sequence using BLAST (NCBI) revealed a single 253 bp region present in most vertebrate species (S1 Fig). Characterization of *LncRNA-HIT ‘s* protein coding potential using the Coding Potential Assessment Tool (CPAT) revealed multiple stop codons in all six reading frames and a protein coding probability of 0.084, well below the 0.44 threshold predicted for protein coding genes (S1 and S2 Figs) (CPAT version 1.2; http://rna-cpat.sourceforge.net, [37]). In comparison, CPAT analysis of neighboring genes, *Hottip* and *Hoxa13*, revealed scores of 0.049 and 0.99 respectively, confirming the poor coding potential of the lncRNA *Hottip* as well as the favorable protein coding potential of *Hoxa13* (S2 Fig). Finally analysis of the potential initiation codons present in the *LncRNA-HIT* transcript also suggested poor protein coding potential, as these sites lack a Kozak consensus sequence and exhibited poor translational potential as determined by the NetStart software package (S2 Fig), http://www.cbs.dtu.dk/services/NetStart/, [38–39].

**Fig 1:**
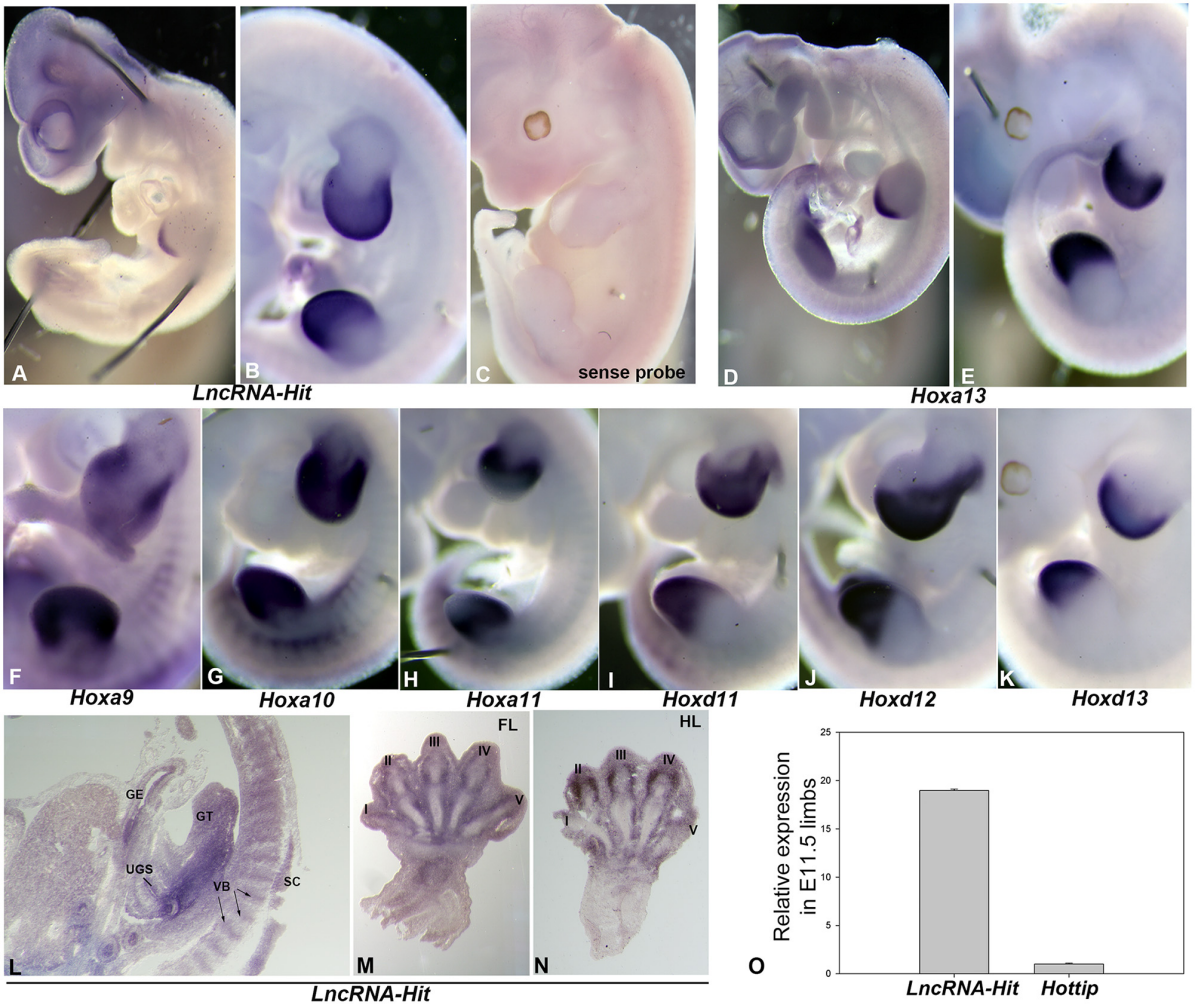
Expression analysis of the lncRNA *LncRNA-HIT*. **(A)** *In situ* hybridization using an antisense *LncRNA-HIT* riboprobe detects the transcript as early as E 10.5 in the distal limb which expands throughout the limb bud at E 11.5 **(B)**. **(C)** *In situ* hybridization using a sense orientation *LncRNA-HIT* riboprobe detects no *LncRNA-HIT* transcripts, confirming the unidirectional transcription of *LncRNA-HIT* in the same orientation as the 5’ HoxA genes in the developing limb. **(D and E)** Analysis of *Hoxa13* expression shows a high level of overlap with *LncRNA-HIT* in the distal limb. **(F-H)** Analysis of *Hoxa9-Hoxa11* expression in the E 11.5 distal limb reveals some overlap with *LncRNA-HIT* in the limb bud. **(I-K)** Analysis of *Hoxd11-Hoxd13* expression in the E 11.5 distal limb reveals some overlap with *LncRNA-HIT*. **(L)** *LncRNA-HIT* is also expressed in the developing genital tubercle, gut epithelium, urogenital sinus, spinal cord, and vertebral bodies at E 13.5. **(M-N)** *LncRNA-HIT* expression is detected in the digit perichondrial tissues, digit joint fields, and in the developing carpal/tarsal skeletal elements in E 13.5 forelimbs and hindlimbs. **(O)** Relative fold expression of *LncRNA-HIT* and *Hottip* expression in the E 11.5 limbs as determined by qRTPCR. Values represent the *Gapdh* normalized average expression of *LncRNA-HIT* and Hottip in the limb calculated from three independent analyses. Bars represent the standard deviation of the mean from the three independent assays. UGS = urogenital sinus, VB = vertebral body, GT = genital tubercle, GE = gut epithelium, SC = spinal cord.

Localization of the *LncRNA-HIT* transcript by *in situ* hybridization revealed expression in the pre-chondrogenic limb mesenchyme as early as embryonic day (E) 10.5 (Fig 1A). By E 11.5 *LncRNA-HIT* expression is expanded to a greater portion of the limb bud, encompassing the majority of the pre-chondrogenic tissues that normally express members of the 5’ HoxA and D gene clusters including: *Hoxa9-13* and *Hoxd11-13* (Fig 1B). By E 13.5, *LncRNA-HIT* expression continued to follow the expression pattern of *Hoxa13*; particularly in the limb perichondrial tissues, gut, genital tubercle, and urogenital sinus (Fig 1L–1N) [27, 40–42]. *In situ* hybridization using the same *LncRNA-HIT* sequence transcribed in the opposite orientation revealed no expression in the limb or other embryonic tissues, confirming reports [43] of its unidirectional transcription in the same orientation as the HoxA genes (Fig 1C, S1 Fig). Finally, quantitation of *LncRNA-HIT* expression in the E 11.0 limb confirmed that it is highly expressed (Ct = 21.4 ± 0.8) at levels nearly nineteen-fold greater than *Hottip*, which resides approximately 5 kb from *LncRNA-HIT* on mouse chromosome 6 (Fig 1O, S1 Fig).

### The *LncRNA-HIT* transcript is localized to the nucleus where it binds p100/CBP complexes

**Fig 2:**
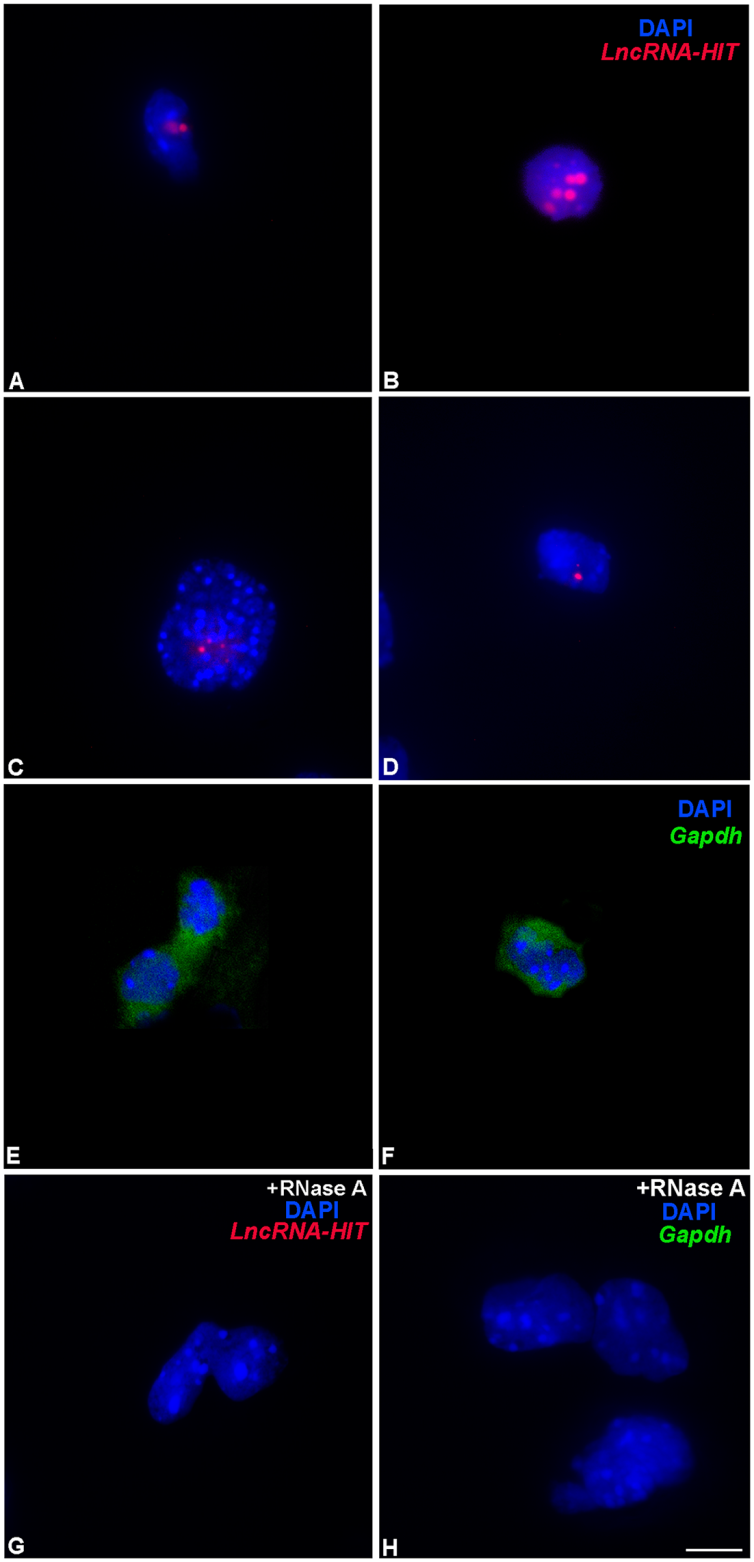
Subcellular localization of *LncRNA-HIT* and *Gapdh* transcripts in limb mesenchyme using single molecule RNA fluorescent in situ hybridization (FISH). **(A-D)** *LncRNA-HIT* transcripts are detected in the nucleus of undifferentiated limb mesenchyme at E 11.0. Red signal = detection of *LncRNA-HIT* probe sets labeled with CalFluor610. Blue signal = DAPI staining of the nuclear DNA. **(E** and **F)** *Gapdh* transcripts are detected in the cytoplasm of undifferentiated limb mesenchyme. Green signal = detection of Gaph probe sets labeled with CalFluor610 and pseudo-colored green. Blue signal = DAPI staining of the nuclear DNA. **(G)** Negative control using RNase A prior to hybridization with the *LncRNA-HIT* probe sets reveals no detected *LncRNA-HIT* (red signal) in the nucleus confirming the detected signal in panels A-D represent hybridization with the *LncRNA-HIT* transcript. The nuclear DNA was unaffected by the RNase A treatment and stained positively with DAPI (blue signal). **(H)** Negative control using RNase A prior to hybridization with the *Gapdh* probe sets reveals no detected *Gapdh* transcript (green signal) in the cytoplasm confirming the detected signal in panels E and F represent hybridization with the *Gapdh transcript*. The nuclear DNA was unaffected by the RNase A treatment and stains positively with DAPI (blue signal). Bar = 10 μm.

A recent analysis of the subcellular localization of 61 lncRNAs revealed a majority localized to the nucleus, recruiting proteins to mediate histone modifications, chromatin accessibility, and gene expression [44]. To gain insight into the potential function of the *LncRNA-HIT*, we first examined its sequence composition, which identified the same nuclear retention motif present in the *BORG* lncRNA [45] (Fig 2, S1 Fig). Analysis of *LncRNA-HIT’s* subcellular localization by single molecule RNA FISH detected the lncRNA in the nucleus where it was distributed diffusely and in larger foci in the limb bud mesenchyme (Fig 2A–2D). In contrast, *Gapdh* transcripts localized primarily to the cytoplasm in these cells, a finding consistent with the protein-coding function of the *Gapdh* transcript (Fig 2E and 2F). Finally, pre-treatment of the fixed limb mesenchyme with RNase A resulted in the complete loss of detection of the *LncRNA-HIT* and *Gapdh* signals, indicating that the signals detected by each FISH probe set corresponds to RNA hybridization rather than hybridization to the corresponding sequence present in the chromosomal DNA. (Fig 2G and 2H).

**Fig 3:**
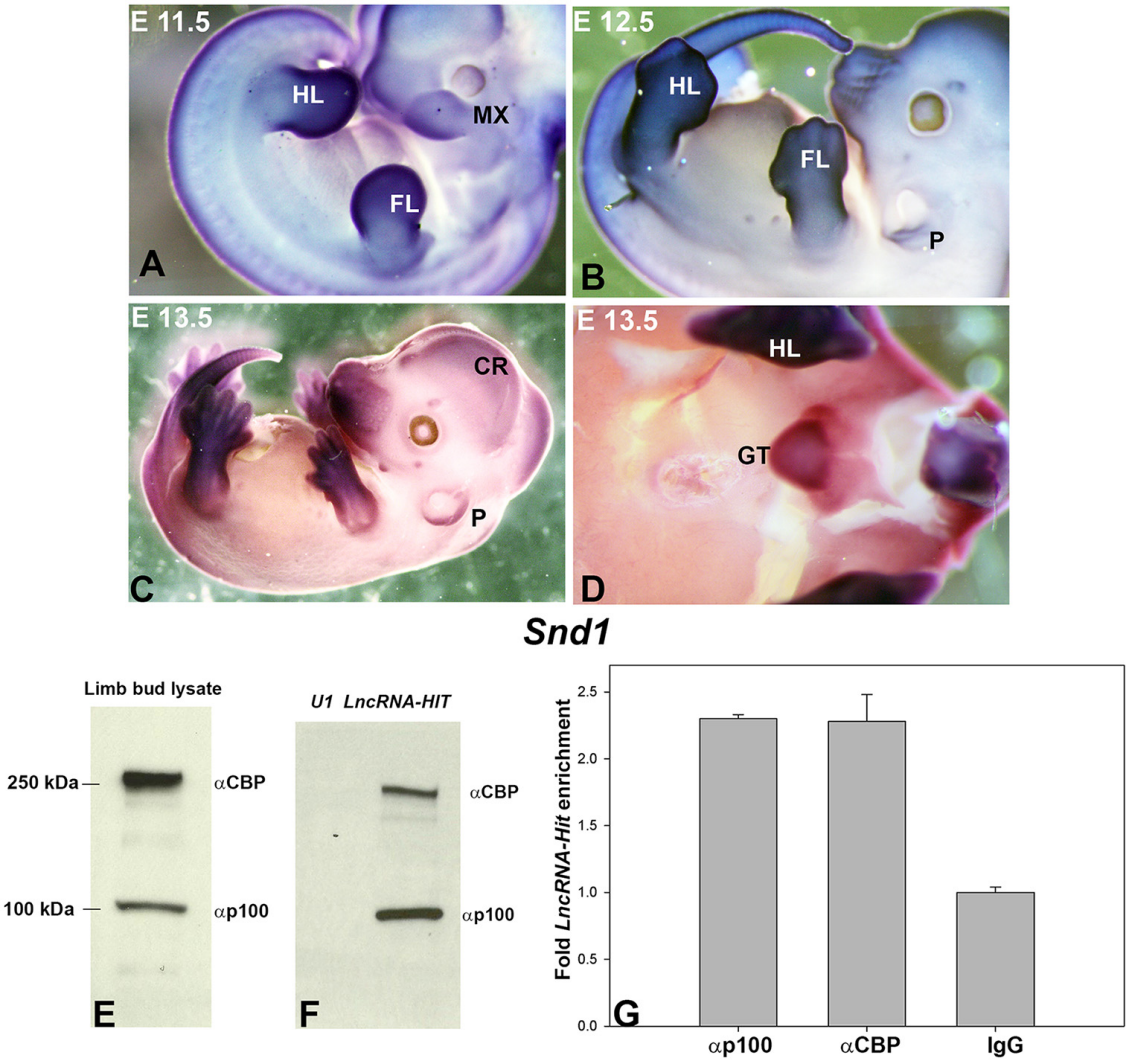
*LncRNA-HIT* recruits a complex of p100 and CBP in the limb. **(A-D)** *Snd1* which encodes p100 is expressed in many of the same regions as *LncRNA-HIT* including the limbs at E11-13.5, spinal cord, and genital tubercle. FL = forelimb, HL = hindlimb, MX = maxillary component of the first branchial arch, SC = spinal cord, P = pinnae. **(E)** Western blot analysis using antibodies specific for CBP and p100 confirm the presence of both proteins in cell lysates from E 11.5 limb buds. **(F)** Streptavidin-mediated precipitation of biotinylated *LncRNA-HIT* and *U1* RNA transcripts reveals co-precipitation of CBP and p100 from limb mesenchyme, confirming recruitment of both proteins by the lncRNA in the limb mesenchyme. **(G)** RIP analysis of p100 and CBP from the limb mesenchyme reveals co-precipitation of endogenous *LncRNA-HIT* transcript, confirming that CBP, p100, and *LncRNA-HIT* are present in a complex in the limb bud mesenchyme. Values represent the mean fold enrichment of *LncRNA-HIT* after precipitation with antibodies specific for CBP, p100 from three independent assays. Control precipitations were performed in parallel using murine IgG. Bars represent the standard deviation of the mean from the three independent assays.

To determine which proteins interact with *LncRNA-HIT* in the nucleus, we first tested whether it functions similarly to its chromosomal neighbor, *Hottip*, which recruits WDR5 to facilitate H3K4me3 [5, 46]. Immunoprecipitation of WDR5 from Flag-WDR5 293T cells transfected with an *LncRNA-HIT* expression vector revealed no enrichment of the *LncRNA-HIT* transcript, suggesting a separate mechanism for *LncRNA-HIT* function in the nucleus (S3 Fig). To identify the proteins binding to *LncRNA-HIT*, an RNA affinity assay was used to isolate limb proteins preferentially binding to the *LncRNA-HIT* transcript. Mass spectroscopy analysis of the U1 control and *LncRNA-HIT* elution fractions identified multiple proteins in the *LncRNA-HIT* and U1 control elution fractions (S4 Fig). As an initial filter, proteins common to both the U1 and *LncRNA-HIT* elution fractions were excluded from subsequent analysis. Several proteins exclusive to the *LncRNA-HIT* elution fractions were also excluded from subsequent analysis as they could not be reproducibly detected in replicate elution fractions. After these initial exclusions, a single protein was identified to be consistently enriched (> 23-fold average enrichment) in only the *LncRNA-HIT* elution fractions. The single protein was identified as p100, a 100 Kd transcriptional co-factor that partners with creb binding protein (CBP) to recruit histone acetyltransferase activity to the STAT6 locus (Fig 3, S4 Fig, and Methods) [47–50]. Analysis of *Snd1* expression which encodes the p100 protein confirmed that p100 is co-expressed with *LncRNA-HIT* in many of the same embryonic regions including the undifferentiated fore- and hindlimb mesenchyme as well as in the developing genital tubercle (Fig 3A–3D). We next evaluated whether p100 and CBP form a complex with endogenous *LncRNA-HIT* in the limb mesenchyme. Western blot analysis of the limb bud proteins co-precipitating with biotinylated-*LncRNA-HIT* revealed enrichment of p100 and CBP, suggesting that both proteins may form a complex with *LncRNA-HIT* (Fig 3E and 3F). Testing this hypothesis, we examined whether endogenous *LncRNA-HIT* present in the limb mesenchyme would co-precipitate with p100 and CBP. Immunoprecipitation of p100 and CBP from limb bud mesenchyme revealed a consistent enrichment of *LncRNA-HIT* (> 2-fold) compared to parallel precipitations using IgG, confirming that the endogenous lncRNA forms a complex with p100 and CBP in the pre-chondrogenic limb mesenchyme (Fig 3G).

### *LncRNA-HIT* contacts multiple chondrogenic loci in the developing limb mesenchyme

The nuclear retention of *LncRNA-HIT* as well as its ability to bind p100/CBP complexes suggested the lncRNA may function at the chromosomal level to modulate gene expression in the developing limb. To identify the chromosomal regions contacted by *LncRNA-HIT*, ChIRP-seq was used to precipitate chromatin fractions associated with the lncRNA from E 11.0 limb bud mesenchyme.

**Fig 4:**
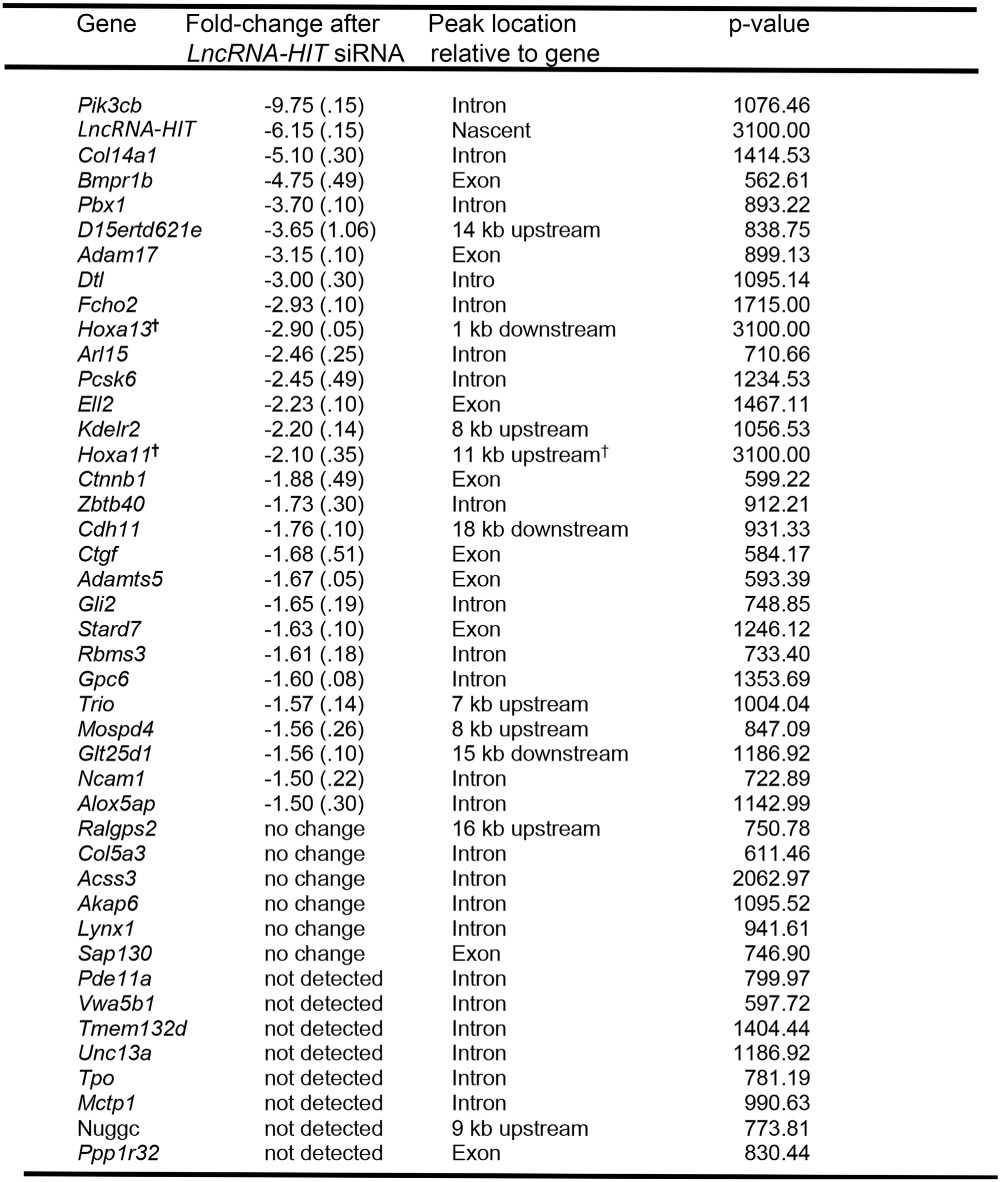
*LncRNA-HIT* peaks are significantly associated with multiple loci which respond to siRNA-mediated reductions in the lncRNA. Values represent mean fold-change decrease in limb bud expression after siRNA treatments compared to scrambled siRNA controls from three independent assays. Standard deviation for these analyses is depicted in parentheses. p-values for the associated peaks is depicted by the transformation -10 log_10_ (p-value) as determined by MACS analysis. †Loci located in *cis* to the nascent *LncRNA-HIT* peak.

The analysis pipeline previously described [51] was used to process the *LncRNA-HIT*-associated peaks as determined by ChIRP-seq. DNA isolated from the precipitated chromatin was submitted to Elim Biopharm (Hayward, CA) for library preparation and next generation sequencing. The sequenced fragments were assembled against murine genome (NCBI37/mm9) using Bowtie and peaks were ranked by MACS using their assigned p-value [52–53]. Visual inspection of the ranked peaks using the UCSC genome browser revealed a marked drop-off in peak signal strength beyond peak 775 (p-value = 5.9 x 10^−57^). Based on this result, the top 775 peaks were selected to identify candidate genes potentially regulated by *LncRNA-HIT*. From the initial cohort of 775 peaks, 173 peaks were excluded from subsequent studies as they were also present in parallel assays using the same *LncRNA-HIT* probe sets and a glial cell line control that does not express *LncRNA-HIT*. The remaining 602 peaks were evaluated for their association with *cis*-regulatory elements in the murine genome using the Genomic Regions of Enrichment of Annotations software (GREAT) (S5 Fig) [54]. Interestingly, while 588 peaks were identified as associating with one or more *cis*-regulatory elements, a correlative function for these associated *cis*-regulatory regions was not identified by the GREAT software package (S5 Fig). Next, because GREAT provides only a computational prediction of function, a second analysis was performed focusing on high confidence peak-to-gene relationships using peaks mapping within 25 kb of a known gene. After this analysis, 42 peak-associated candidate genes were identified as potential targets for regulation by *LncRNA-HIT-*p100/CBP complexes (Fig 4). To validate the regulation of these genes by *LncRNA-HIT-*p100/CBP complexes, E 11.0 limb mesenchymal cells were transfected with two siRNAs specific for the *LncRNA-HIT* transcript (-6.15-fold knockdown, Fig 4) and evaluated for changes in gene expression in three independent assays using qRTPCR. From this analysis, 28 near-peak genes exhibited decreased expression in response to the *LncRNA-HIT* siRNA treatments with fold-change differences ranging from -1.5- to -9.75 compared to transfections using scrambled siRNA controls (Fig 4). The remaining near-peak genes exhibited no change in expression in response to the *LncRNA-HIT* siRNA treatments (6 genes) or could not be detected in the E 11.0 limb mesenchyme by qRTPCR (8 genes) (Fig 4). The 28 genes exhibiting decreased expression in response to the *LncRNA-HIT* siRNA treatments were queried as a group for ontological function using the AmiGO 2 term enrichment software which identified proximal/distal pattern formation and regulation of chondrocyte differentiation (p≤ 0.05) as the most significant ontological categories, suggesting a role for *LncRNA-HIT* in the regulation of genes required for limb chondrogenesis (S6 Fig, and Methods).

**Fig 5:**
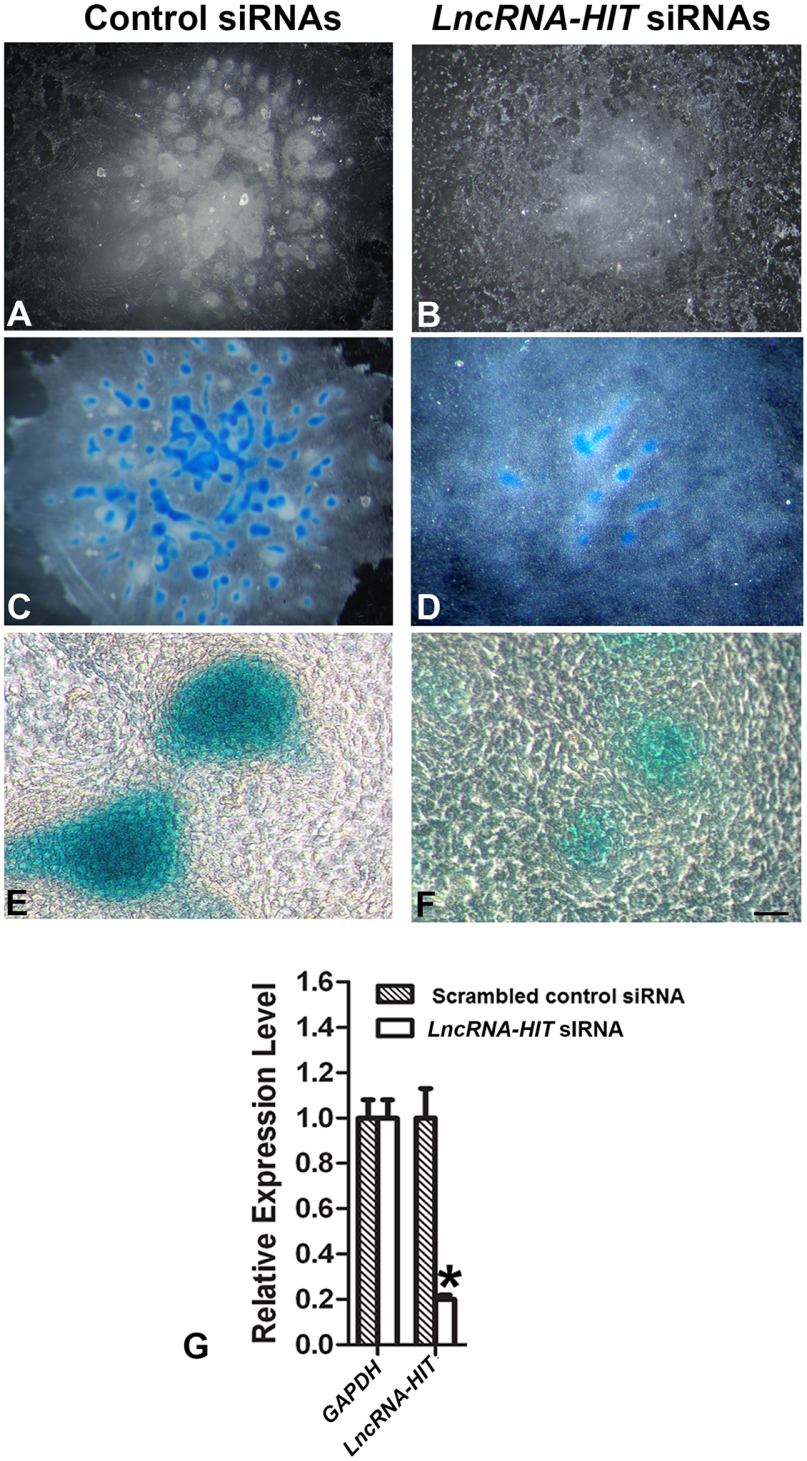
Chondrogenic differentiation is impacted by siRNA-mediated reductions in *LncRNA-HIT* in micromass assays. **(A)** Mesenchymal cell condensation is unaffected after transfection of the dissociated limb mesenchyme with a scrambled siRNA control. **(C and E)** After condensation, the scrambled control transfected limb mesenchyme are competent to differentiate into cartilage nodules that stain positively for the cartilage marker alcian blue. **(B,D,F)** Parallel assays transfecting the same limb mesenchyme with two siRNAs specific for *LncRNA-HIT* result in a loss in cell condensation (panel B) as well as severe reductions in the formation of cartilage nodules that stain with alcian blue (panels D and F). **(G)** Quantitation of the *LncRNA-HIT* levels 24H after transfection with the *LncRNA-HIT* siRNAs or scrambled siRNAs reveal an eighty percent reduction in endogenous *LncRNA-HIT* levels compared to parallel transfections using a scrambled siRNA control. Values represent the average relative expression calculated from six independent replicates. Bars represent the standard deviation of the mean from the six independent assays. A Student’s t-test was used to determine significance of the averaged *LncRNA-HIT* expression values compared to the scrambled *LncRNA-HIT* siRNA control. A significant difference is indicated by an asterisk.

To test whether *LncRNA-HIT* regulates chondrogenesis in the limb mesenchyme, an *in vitro* micromass chondrogenesis assay was used, as it recapitulates many of the cellular events occurring during chondrogenesis including mesenchymal cell condensation, cartilage extracellular matrix expression, and cartilage nodule formation and is amenable to siRNA-mediated knockdown of pro-chondrogenic genes including *Runx1*, *Ctgf*, *Notch*, and *Angptl4* [27, 55–62]. Transfection of limb mesenchyme with siRNAs specific for *LncRNA-HIT* significantly reduced *LncRNA-HIT* RNA levels by nearly eighty percent, confirming the effectiveness of the siRNAs to target *LncRNA-HIT* for degradation in the micromass assay (n = 6 independent replicates, Fig 5). Analysis of the micromass assays transfected with the *LncRNA-HIT* siRNAs revealed a substantial reduction in chondrogenesis, resulting in the formation of fewer cartilage nodules that stained with alcian blue (n = 6 independent assays, Fig 4). In contrast, parallel micromass assays using the same populations of limb mesenchyme cells transfected with a scrambled control siRNA confirmed that the transfected limb mesenchyme was competent to undergo chondrogenic differentiation, exhibiting robust cell condensation and the formation of multiple alcian blue positive cartilage nodules (n = 6 independent assays, Fig 5).

**Fig 6:**
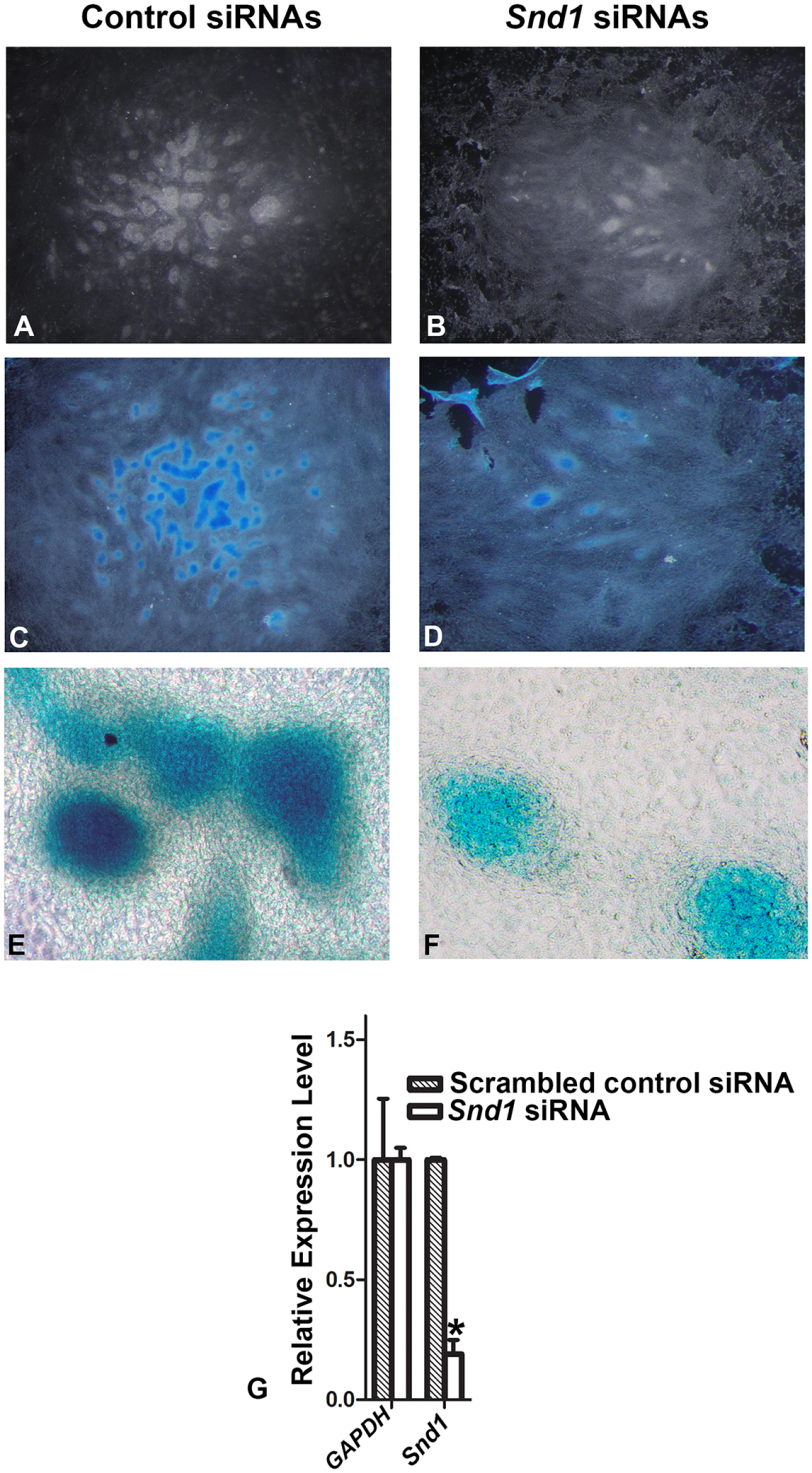
Chondrogenic differentiation is impacted by siRNA-mediated reductions in Snd1 (p100) in micromass assays. **(A)** Mesenchymal cell condensation is unaffected after transfection of the dissociated limb mesenchyme with a scrambled siRNA control. **(C and E)** After condensation, the scrambled control transfected limb mesenchyme are competent to differentiate into cartilage nodules that stain positively for the cartilage marker alcian blue. **(B,D,F)** Parallel assays transfecting the same limb mesenchyme with a *Snd1* siRNA cocktail severely impacts mesenchymal cell condensation producing few pre-cartilaginous condensations that can differentiate into mature cartilage nodules that stain positively with alcian blue. **(G)** Quantitation of the *Snd1* levels 24H after transfection with the *Snd1 siRNA cocktail* or scrambled siRNA controls reveal a seventy percent reduction in endogenous *Snd1* levels compared to parallel transfections using the scrambled siRNA controls. Values represent the average relative expression calculated from six independent replicates. Bars represent the standard deviation of the mean from the six independent assays. A Student’s t-test was used to determine significance of the averaged *Snd1* expression values compared to the scrambled *s*iRNA controls. A significant difference is indicated by an asterisk.

Next, if chondrogenesis is facilitated by *LncRNA-HIT’s* recruitment of p100/CBP complexes, then siRNA-mediated reduction in *Snd1*, which encodes p100, should also affect the chondrogenic capacity of the undifferentiated limb mesenchyme. Testing this hypothesis, micromass assays using limb mesenchyme transfected with *Snd1* siRNAs produced a similar loss in *Snd1* transcript levels (75% knockdown) and reduced chondrogenesis, resulting in the formation of few cartilage nodules staining with alcian blue (n = 6 independent assays, Fig 6). Control transfections using a scrambled *Snd1* siRNA consistently resulted in robust cartilage formation producing numerous cartilage nodules staining positively for alcian blue (n = 6 independent assays, Fig 5), supporting the hypothesis that *LncRNA-HIT* and its recruited proteins are essential for chondrogenic differentiation of the early limb mesenchyme.

### *LncRNA-HIT* exhibits enhancer RNA function to promote H3K27ac to facilitate gene expression

**Fig 7:**
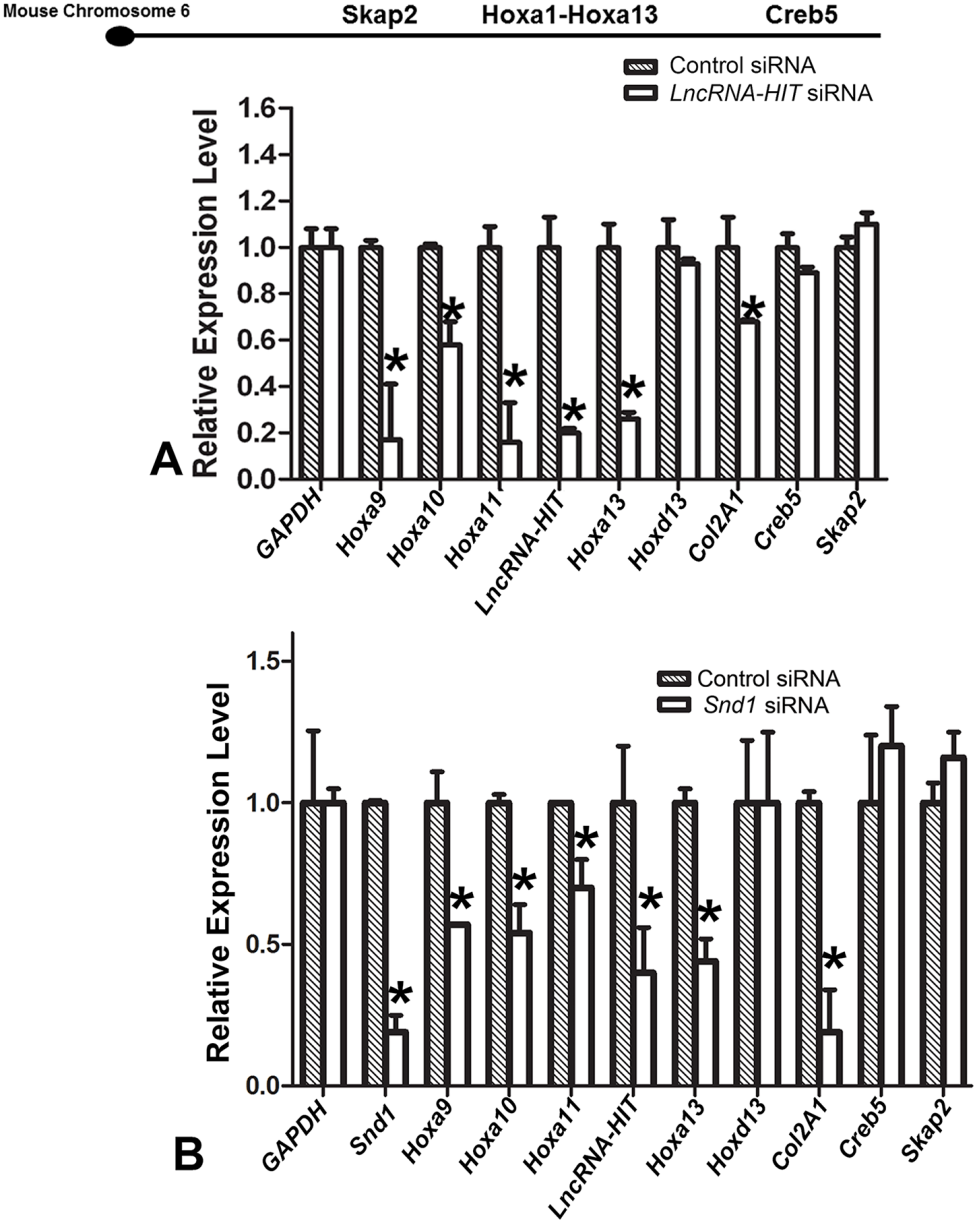
siRNA-mediated reduction in *LncRNA-HIT* or *Snd1* results in reduced levels of 5’ HoxA gene expression. **(A)** Relative gene expression after transfection with *LncRNA-HIT* siRNAs or scrambled control siRNAs. Values represent average expression levels calculated from six independent replicates. Bars represent the standard deviation of the mean from the six independent replicates. Asterisks denote a significant changes in gene expression as determined a Student’s t test. **(B)** Relative gene expression after transfection with *Snd1* siRNAs or scrambled control siRNAs. Values represent average expression levels calculated from six independent replicates. Bars represent the standard deviation of the mean from the six independent replicates. Asterisks denote a significant changes in gene expression as determined a Student’s t test.

The decrease in *Hoxa13* and *Hoxa11* expression in response to the *LncRNA-HIT* siRNA treatments (Fig 4) suggested that *LncRNA-HIT* may be functioning as an enhancer RNA, using its recruitment of p100/CBP to its site of transcription to promote neighboring 5’ HoxA gene expression through its maintenance of H3K27ac. Testing this hypothesis, we first examined whether siRNA-mediated reductions in *LncRNA-HIT* or *Snd1* impact expression of additional genes proximal to the nascent site of *LncRNA-HIT* transcription. qRTPCR analysis of limb mesenchyme treated with *LncRNA-HIT-* or *Snd1* (p100)-specific siRNAs revealed significant reductions in expression for all 5’HoxA gene members (Fig 7), suggesting that *LncRNA-HIT* is functioning as an enhancer lncRNA to mediate expression of the 5’ HoxA genes. Interestingly, no changes in expression were detected for *Creb5* and *Skap2*, which flank the HoxA cluster, and for *Hoxd13*, suggesting a specific effect on the 5’ HoxA genes which are proximal to the site of *LncRNA-HIT* transcription (Fig 7). Changes in 3’ HoxA gene expression (*Hoxa1-Hoxa7*) could not be determined, as their expression in the limb mesenchyme is below the level of detection by qRTPCR. Finally *Col2a1*, a strong indicator of chondrogenic differentiation, also exhibited reduced expression in response to the *LncRNA-HIT* and *Snd1* siRNA treatments confirming the disruption in chondrogenesis exhibited by the micromass assays (Figs 5–7).

**Fig 8:**
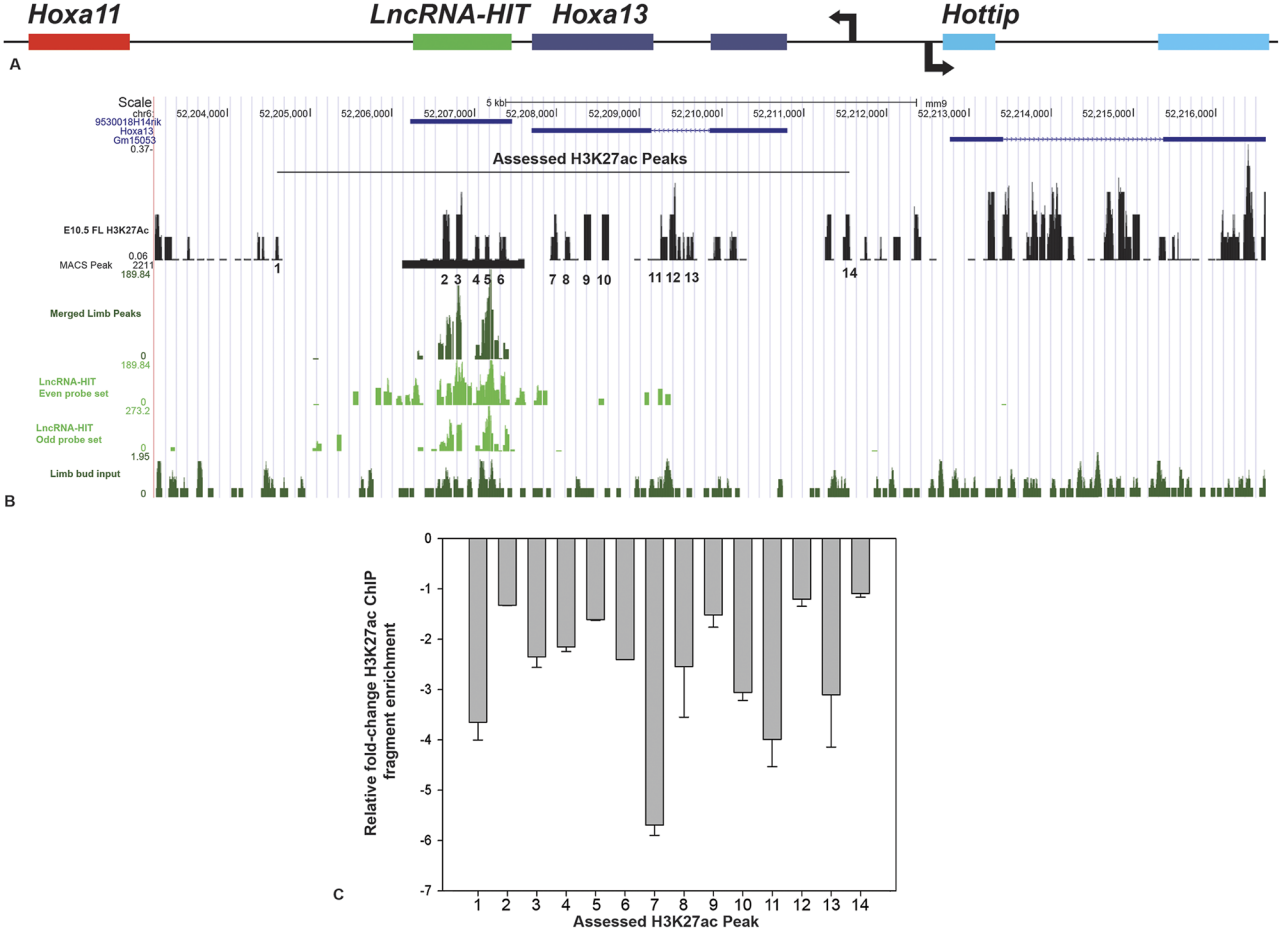
siRNA-mediated loss of *LncRNA-HIT* affects H3K27ac at its site of nascent transcription. **(A)** Relative location of *LncRNA-HIT* and its nearest neighbors, *Hoxa11* and *Hoxa13*. **(B)** Visualization of the nascent *LncRNA-HIT* (green peaks) detected by ChIRP-seq using the USCS Genome Browser. Peaks identified by the even and odd *LncRNA-HIT* probe sets are indicated on the left axis using even and odd probe sets specific for the *LncRNA-HIT* lncRNA and displayed by the UCSC genome browser. The localization of the fourteen H3K27ac peaks (black peaks) proximal to the *LncRNA-HIT* associated peaks as determined by overlaying the H3K27ac limb bud ChIP-seq dataset, GSE3064 [62]. **(C)** qChIP Analysis of the fourteen H3K27ac regions proximal to the nascent *LncRNA-HIT* peaks revealed a loss in fragment enrichment in response to the *LncRNA-HIT* siRNA treatments in limb bud mesenchyme. Values represent the mean fold change H3K27ac fragment enrichment compared to parallel treatments using scrambled *LncRNA-HIT* siRNA controls for three independent assays. Error bars represent the standard deviation of the mean for the three independent assays.

Next we hypothesized that H3K27ac should also be affected by siRNA-mediated reductions in *LncRNA-HIT* as CBP recruitment would be concomitantly affected in the limb mesenchyme. Mapping of the H3K27ac-tagged chromosomal regions in the E 10.5 limb was previously reported (GEO Dataset: GSE30641, [63]). Using this dataset, the H3K27ac sites proximal to the *LncRNA-HIT* -associated loci were examined for changes using an acetylation-specific H3K27 antibody and quantitative chromatin immunoprecipitation (qChIP) (Fig 8). Starting with the site of nascent *LncRNA-HIT* transcription, qChIP analysis of fourteen H3K27ac-tagged chromosomal regions between *Hoxa11* and *Hoxa13* revealed consistent reductions in precipitated fragment enrichment in response to the *LncRNA-HIT* siRNA treatments (n = 3 independent assays) (Fig 7). Most notable was a greater than five-fold reduction in H3K27ac fragment enrichment for the region associated with exon 2 of *Hoxa13* (Fig 8, fragment 7). A greater than 3-fold decrease in H3K27ac chromatin fragment enrichment was also detected in the *Hoxa13* intronic region (Fig 8, fragment 11) and in chromatin fragments more proximal to *Hoxa11* (Fig 8, fragment 1). Several H3K27ac regions were also identified within the *LncRNA-HIT* locus (Fig 8B). qChIP analysis of these regions revealed reduced enrichment for the entire region, with fold change decreases ranging from 1.5 to 2.8 (Fig 8, fragments 2–6).

**Fig 9:**
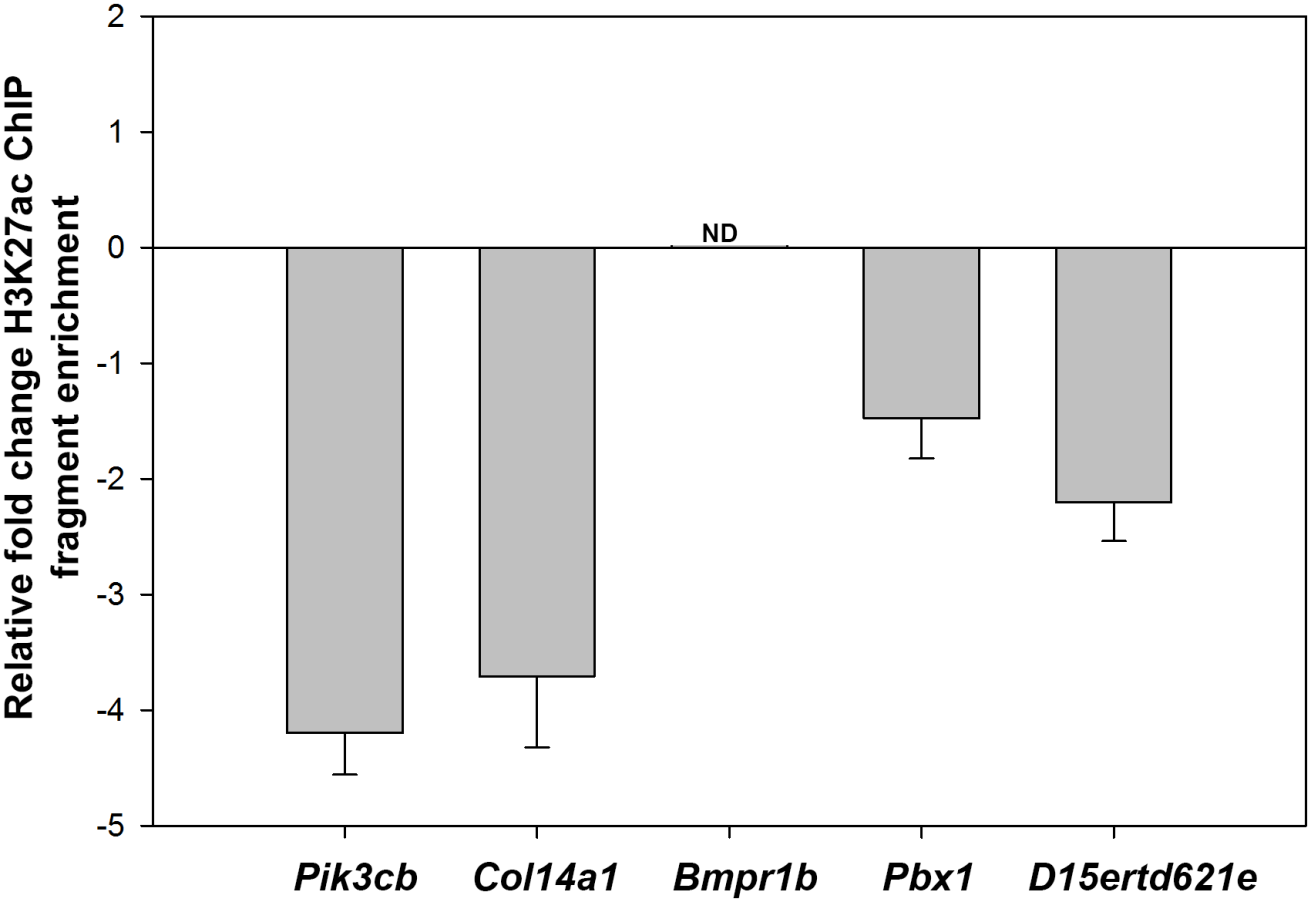
qChIP analysis of H3K27ac at the top five loci impacted by the *LncRNA-HIT* siRNA treatments. Values represent the mean fold change H3K27ac fragment enrichment compared to parallel treatments using scrambled *LncRNA-HIT* siRNA controls for three independent assays. Error bars represent the standard deviation of the mean for the three independent assays. ND = no qPCR product detected for the H3K27ac peak proximal to *Bmpr1b*.

Expanding this analysis, we examined additional chromosomal loci bound by *LncRNA-HIT* for changes in H3K27ac fragment enrichment in response to the siRNA treatments. For this analysis, we selected the H3K27ac sites most proximal to five loci exhibiting the greatest decrease in expression in response to the *LncRNA-HIT* siRNA treatments: *Pik3cb*, *Col14a1*, *Bmpr1b*, *Pbx1*, and *D15ertd621e* (Fig 4). qChIP analysis revealed consistent reductions in immunoprecipitated H3K27ac fragments for four of the five candidate loci (n = 3 independent assays) including *Pik3cb* (> 4-fold decrease), *Col14a1* (> 3-fold decrease), *Pbx1* (>1.4-fold decrease) and *D15ertd621e* (>2-fold decrease) (Fig 9). Attempts to amplify the immunoprecipitated H3K27ac fragments proximal to the *LncRNA-HIT* associated region at the *Bmpr1b* locus were unsuccessful using several primer pair combinations and sheared chromatin from the *LncRNA-HIT—*or control-siRNA treated samples, suggesting that H3K27ac in this region is insufficient to facilitate immunoprecipitation by the acetylation-specific H3K27 antibody (Fig 9).

**Fig 10:**
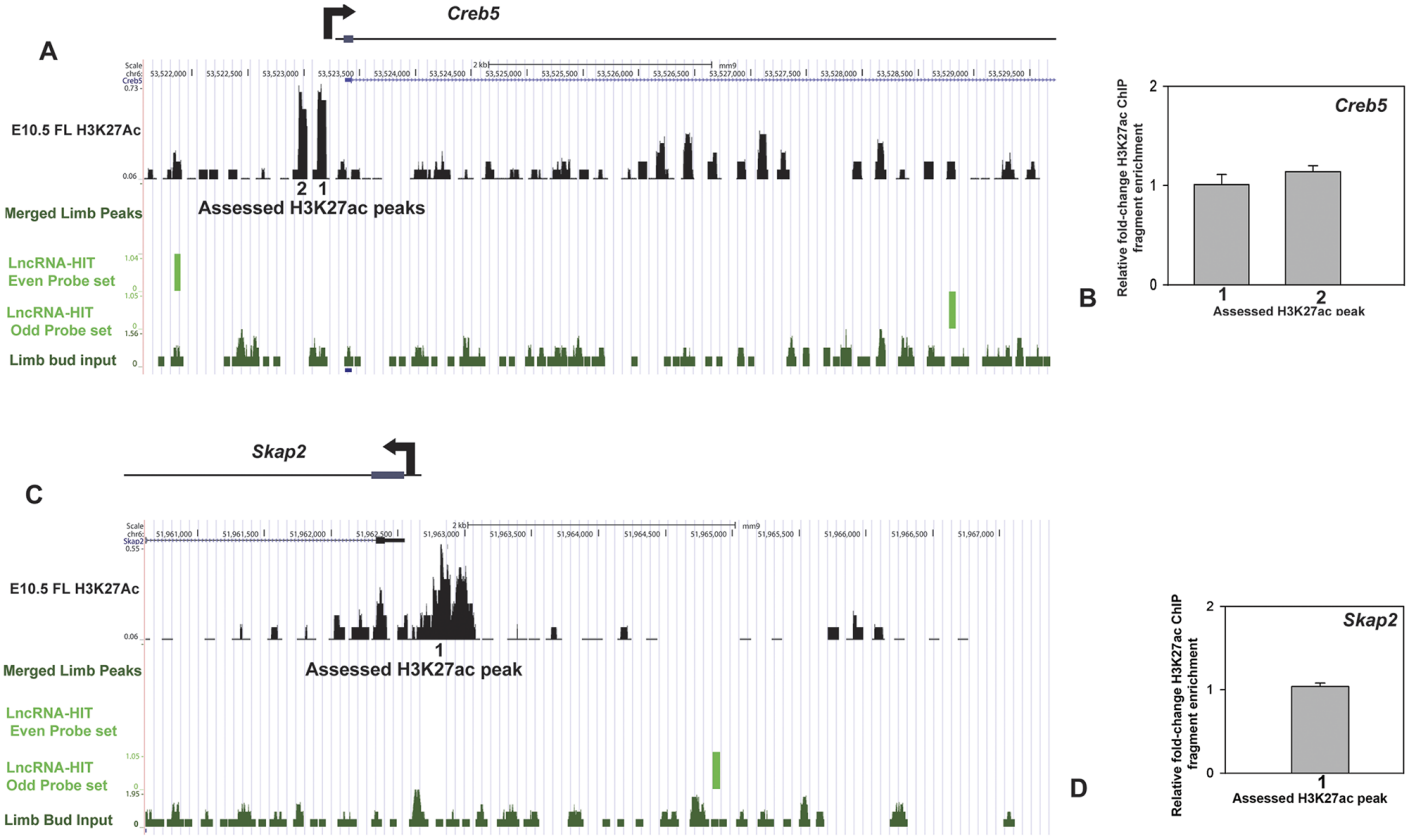
qChIP analysis of H3K27ac at *Creb5* and *Skap2* which lack LncRNA-HIT associated peaks. **(A)** *Creb5*-assocaited H3K27ac peaks assessed for changes in fragment enrichment in response to LncRNA-HIT siRNA treatments. **(B)** qChIP analysis of H3K27ac peak enrichment in response the LncRNA-HIT siRNA treatments at the *Creb5* locus. Values represent the mean fold change H3K27ac fragment enrichment relative to parallel treatments using a scrambled siRNA control. Bars represent the standard deviation of the mean for three independent assays. **(C)** *Skap2*-associated H3K27ac peak assessed for changes in fragment enrichment in response to LncRNA-HIT siRNA treatments. (**D**) qChIP analysis of H3K27ac peak enrichment in response the LncRNA-HIT siRNA treatments at the *Skap2* locus. Value represents the mean fold change H3K27ac fragment enrichment relative to parallel treatments using a scrambled siRNA control. Bar represents the standard deviation of the mean for three independent assays.

Finally to test whether changes in H3K27ac peak enrichment was specifically affected by the *LncRNA-HIT* siRNA treatments we examined loci lacking associated *LncRNA-HIT* peaks for changes in H3K27ac fragment enrichment. Analysis of H3K27ac fragments associated with the promoter regions of *Creb5* and *Skap2* revealed no changes in fragment enrichment in response to *LncRNA-HIT* siRNA treatments, supporting the conclusion that *LncRNA-HIT*-p100/CBP is functioning to maintain H3K27ac status in regions bound by the lncRNA-protein complex (Fig 10).

### *LncRNA-HIT*and p100 cooperate to promote gene expression

**Fig 11:**
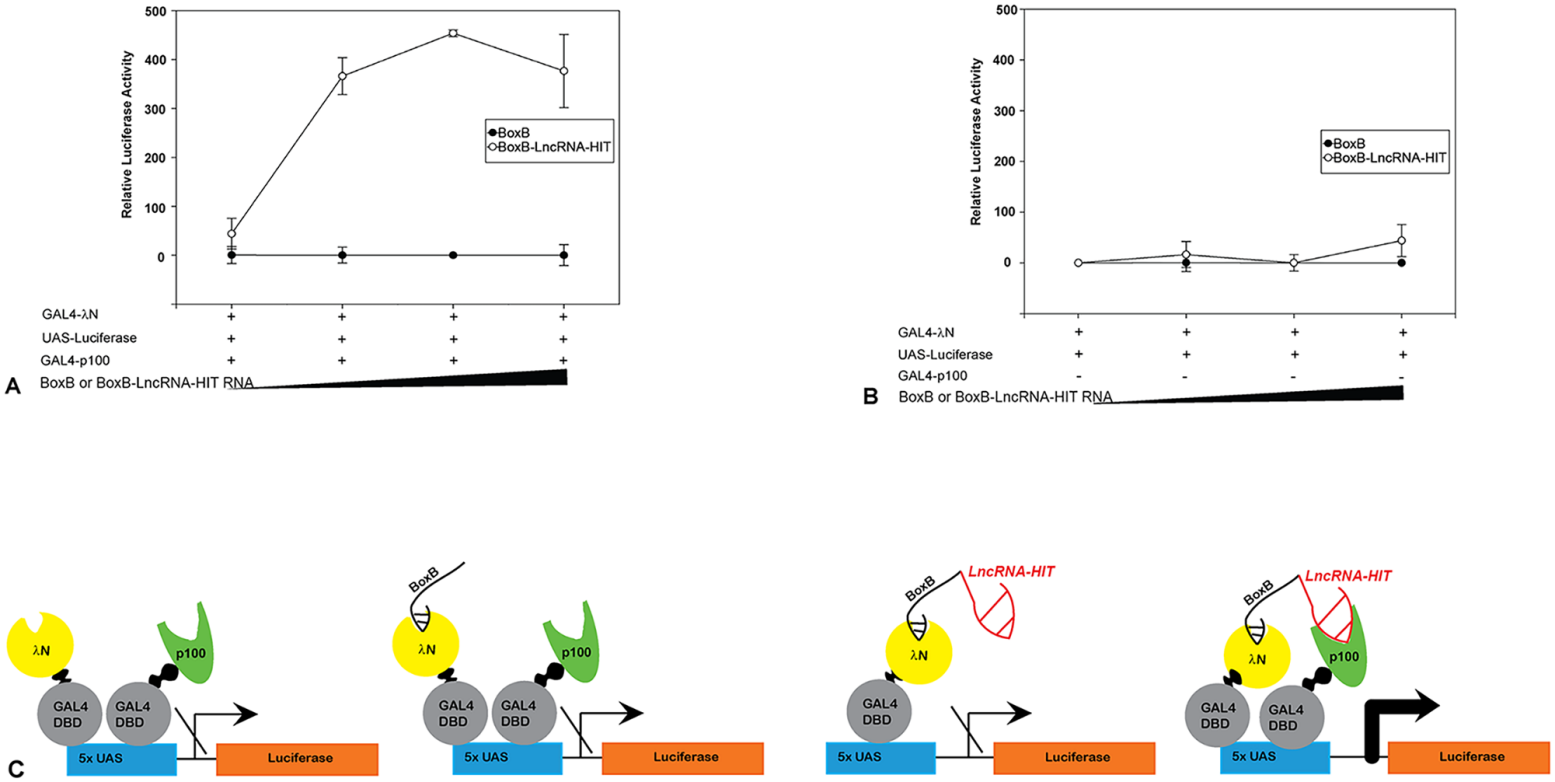
Co-recruitment of *LncRNA-HIT* and p100 is required to stimulate gene expression from a synthetic locus. **(A)** Analysis of UAS-luciferase reporter activation after GAL4-λN-BoxB*LncRNA-HIT* and GAL4-p100 recruitment. **(B)** Analysis of UAS-luciferase reporter activation in the absence of GAL4-p100. **(C)** Model of UAS-luciferase reporter activation in response to the recruitment of *LncRNA-HIT* and p100. For panels A and B, increasing dosages (0, 50, 100, 250, ng) of the BoxB or BoxB-*LncRNA-HIT* transcripts are represented by the black triangle.

We next examined whether the recruitment of p100 by *LncRNA-HIT* contributes to the regulation of near-peak gene expression. To address this question, an RNA tethering assay was used to dissect the gene-regulatory contributions of *LncRNA-HIT* and p100 towards the activation of a synthetic UAS-luciferase reporter (Fig 11). Recruitment of *LncRNA-HIT* to the reporter locus was facilitated by adding five copies of the *BoxB* transcript to the *LncRNA-HIT* RNA. The *BoxB* transcript is strongly bound by the RNA binding protein λN, which when fused to the GAL4 DNA binding domain facilitates the recruitment of the *BoxB*-*LncRNA-HIT* transcript to the UAS luciferase locus (Fig 11) [5,64]. Co-transfection of the UAS-luciferase reporter, λN-GAL4, and p100-GAL4 expression vectors resulted in no activation of the UAS luciferase reporter, indicating that the recruitment of these factors to the synthetic UAS locus is not sufficient to activate gene expression (Fig 11). Similarly, co-transfection of the UAS-luciferase reporter and expression vectors encoding λN-GAL4, p100-GAL4, and the *BoxB* transcript lacking *LncRNA-HIT* also resulted in no activation of the UAS luciferase reporter, indicating that the recruitment of *BoxB*, p100, and λN to the synthetic locus is not sufficient to activate gene expression (Fig 11A and 11C). Additionally, luciferase expression was not detected in cells transfected with UAS-luciferase reporter and expression vectors encoding *BoxB-LncRNA-HIT* and λN-GAL4, suggesting that the recruitment of *LncRNA-HIT* to the UAS locus is not sufficient to activate gene expression (Fig 11B and 11C). Finally, co-transfections using the UAS-luciferase reporter, *BoxB*-*LncRNA-HIT*, λN-GAL4, and p100-GAL4 expression vectors resulted in strong activation of the luciferase reporter in a dosage-dependent manner, indicating that co-recruitment of p100 and *LncRNA-HIT* to a locus is required to activate gene expression (Fig 11A and 11C).

## Discussion

The development of the vertebrate limb requires a coordinated series of cellular events to facilitate the formation of bone and articular cartilage. Initiating this process is the proliferation and migration of mesenchymal progenitor cells from the lateral plate mesoderm. Once in the limb bud, the mesenchymal cells condense forming cartilage templates for all skeletal tissues. Finally the chondrocytes within these templates mature, forming bone through endochondral ossification or remain as a specialized population of chondrocytes to form the articular cartilage [65–68]. Interestingly, while SOX9 appears to be essential for many of the cellular processes required for skeletal development, emerging evidence suggests that an epigenetic component, mediated by lncRNAs, may also play an important role in the formation, maintenance, or pathology of the appendicular skeletal tissues [5,34,69–73]. Given the large number of lncRNAs expressed by the human (>15,000) and mouse genomes (>7600), it is likely that additional roles for these molecules will be identified in the appendicular cartilage and bone (GENCODE ver. 22, [74]). In this report, we identify the lncRNA, *LncRNA-HIT*, as an essential component for chondrogenesis, regulating multiple genes to facilitate the formation of cartilage from undifferentiated limb mesenchyme.

### *LncRNA-HIT* regulates BMP signaling to facilitate chondrogenesis

Ontological analysis of loci contacted and regulated by *LncRNA-HIT* suggests that the lncRNA coordinates the expression of genes whose products mediate chondrogenic differentiation in the limb mesenchyme. This conclusion is consistent with the decrease in cartilage nodule formation in response to the *LncRNA-HIT* and *Snd1* siRNA treatments. Early studies of cartilage formation identified disruptions in the initial condensation of the limb mesenchyme as a primary cause of reduced cartilage formation [75–76]. More importantly the regulation of mesenchymal condensation was recently shown to require BMP-SMAD4 signaling, independent of SOX9 function [77]. This finding provides a mechanism to explain the loss of cartilage formation in the micromass assays, as *Bmpr1b*, a major receptor for GDF5 and other BMPs during limb development, is significantly down-regulated in response to the *LncRNA-HIT* siRNA treatments (Fig 4) [78–80]. In the absence of BMPR1B, the primary chondrogenic phenotype exhibited by humans and mice is the loss of prechondrogenic condensations and skeletal element hypoplasia, particularly in the distal limb, a region that also strongly expresses *LncRNA-HIT* (Fig 1) [80–83]. Reductions in *Bmpr1b* expression in response to the *LncRNA-HIT* siRNA treatments could also reflect a loss in p100 enrichment. This conclusion is supported by the RNA tethering assay which determined that interactions between *LncRNA-HIT* and p100 were sufficient to activate gene expression from a synthetic locus. By this mechanism, *LncRNA-HIT* could regulate *Bmpr1b* as well as additional loci by stabilizing p100 at the *Bmpr1b* locus; providing a functional explanation for the 4.75-fold decrease in *Bmpr1b* expression in response to the *LncRNA-HIT* siRNA treatments. Alternatively, the down-regulation of *Bmpr1b* expression in response to the *LncRNA-HIT* siRNA treatments could also reflect indirect regulation of *Bmpr1b* by another *LncRNA-HIT*-regulated gene product.

A second component contributing to the control of chondrogenesis by *LncRNA-HIT* is its regulation of *Hoxa13 and Hoxa11* which previous studies indicate can regulate additional components of the BMP-signaling cascade including *Bmp2* and *Bmp7* by HOXA13 and *Runx2 by* HOXA11 [84–85]. Thus in the absence of *LncRNA-HIT* function, the expression of receptor, ligand, and transcriptional components of the BMP signaling pathway are reduced, which in combination, explains the reductions in mesenchymal cell condensation and cartilage nodule formation exhibited by the micromass assays treated with the *LncRNA-HIT* siRNAs.

### *LncRNA-HIT* functions as an enhancer lncRNA

**Fig 12:**
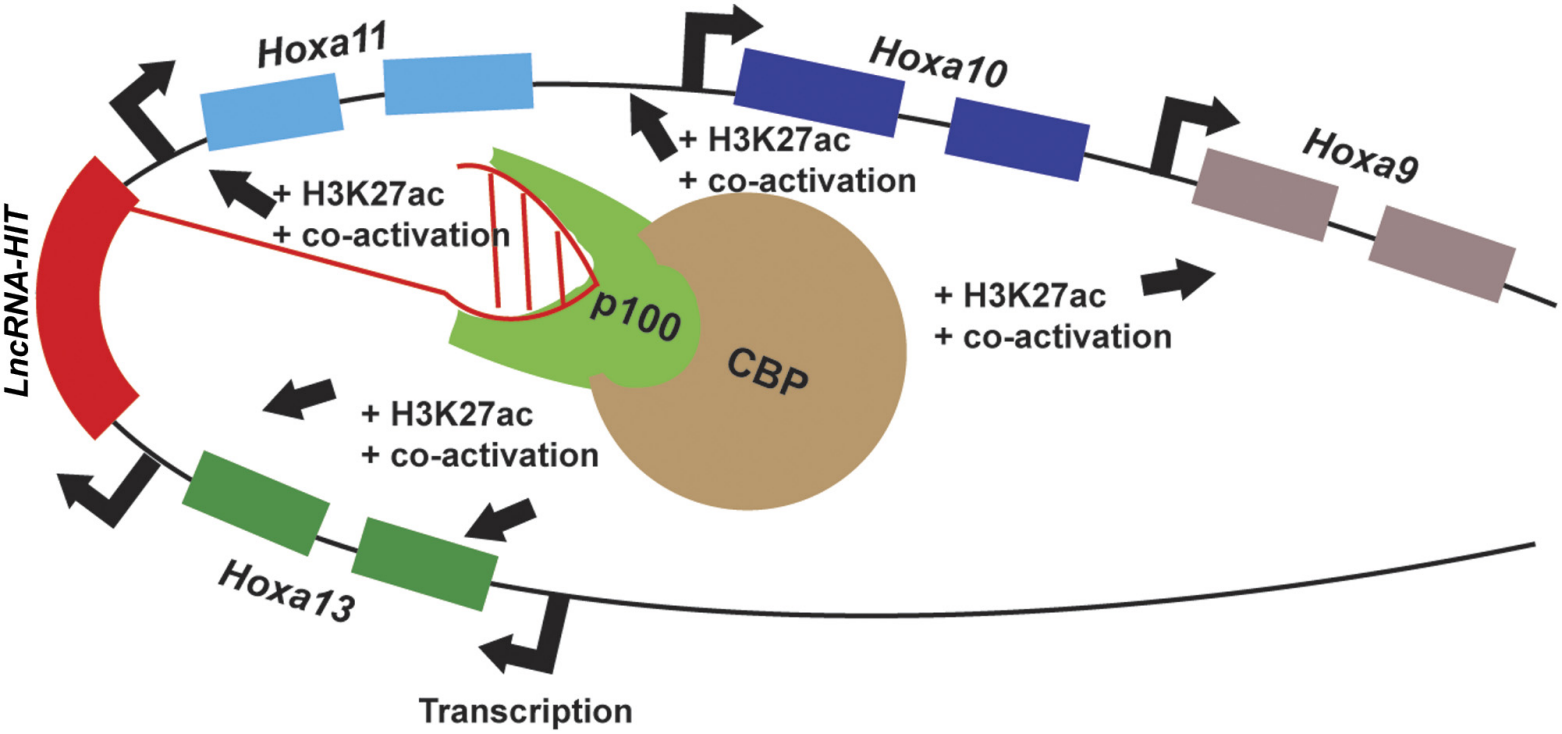
Model of enhancer RNA function of *LncRNA-HIT* at the 5’ HoxA locus.

The down-regulation of 5’ HoxA genes in response to *LncRNA-HIT* siRNA treatments suggests that *LncRNA-HIT* may be functioning as an enhancer lncRNA. Recent studies of enhancer lncRNAs indicate that these molecules regulate the expression of nearby genes by recruiting chromatin modifying proteins to increase the accessibility of the chromosomal region to gene-regulatory factors [6,22,86]. In the limb mesenchyme, our detected decrease in H3K27ac and 5’ HoxA gene expression in response to *LncRNA-HIT* siRNA treatments support a role for *LncRNA-HIT* as an enhancer lncRNA using its associated proteins (p100 and CBP) to facilitate expression of the surrounding 5’ HoxA genes (Fig 12). It is interesting to speculate that the presence of *LncRNA-HIT* within the 5’ HoxA cluster in many vertebrate genomes (S1 Fig) may reflect its conservation as an essential enhancer of 5’ HoxA gene expression, providing a fundamental epigenetic mechanism to promote chondrogenic differentiation; presumably through its recruitment of the p100/CBP complexes. P100/CBP complexes are important components of the STAT6-dependent enhanceosome which functions as a transcriptional enhancer of genes involved in the interleukin 4 gene regulatory cascade [47–48,87]. In limb mesenchyme, *LncRNA-HIT* may be working in a similar capacity, forming a complex with p100 and CBP to regulate pro-chondrogenic gene expression.

Interestingly, the results from the RNA tethering assay also suggest that interactions between *LncRNA-HIT* and p100 may be required to confer functional competence to the RNA protein complex, as the activation of the luciferase reporter occurred only when p100 and *LncRNA-HIT* were co-expressed. Recent studies support this conclusion, as mutations in WDR5 that prevent lncRNA binding impacts WDR5-MLL chromatin occupancy, maintenance of H3K4 trimethylation, and the regulation of genes necessary for embryonic stem cell self-renewal [46]. Taken together these findings suggest that *LncRNA-HIT* promotes gene expression by two mechanisms, first: by binding to p100 to stabilize the protein to promote its co-activator function and second: through its recruitment of CBP to promote chromatin accessibility by maintaining H3K27ac which in the 5’ HoxA region facilitates the expression of multiple factors whose products instruct the limb mesenchyme to form skeletal tissues [27, 30–31,42, 88–89].

### *LncRNA-HIT* may have additional functions in onset and progression of some cancers and in the development of other tissues

Transcriptional profiling of breast tumor cell lines initially identified *LncRNA-HIT* as a TGFβ-induced transcript [35]. Interestingly, TGFβ1/SMAD signaling can also promote *Snd1* (p100) expression which can facilitate epithelial to mesenchymal transitions (EMT) in mammary tumor cell lines, a causal event in tumor cell migration and invasion [90–94]. In these tissues, Tgfβ should induce both *LncRNA-HIT* and *Snd1* (p100) which would activate *Hoxa13*, which has been previously shown to mediate the formation of vascular tissues, providing a mechanism to promote tumor vascularization and growth [95].

Interestingly several studies investigating predicative biomarkers of cancer progression and patient outcomes in hepatocellular carcinoma have implicated the expression *HOXA13* and *HOTTIP* as indicators of poor prognosis with high disease progression [96–97]. Given that the physical distance between *LNCRNA-HIT*, *HOXA13*, and *HOTTIP* is only 6.5 kb in both humans and mice, it is possible that *LNCRNA-HIT’s* function may also be co-opted in these cancers. If human *LNCRNA-HIT* is determined to be expressed in hepatocellular carcinoma, a ChIRP-seq approach could be used to determine the loci contacted by the lncRNA-protein complex, providing additional therapeutic targets for this disease. Moreover, several non-chondrogenic regions also express *LncRNA-HIT* during murine development including the gut epithelium and the genital tubercle, suggesting that the lncRNA may have additional roles in the formation of these tissues. Using the approaches outlined in this study, including ChIRP-seq and RNA affinity chromatography the proteins interacting with *LncRNA-HIT* in these tissues as well as the loci contacted by the protein-lncRNA complexes can be identified providing new insights into the developmental regulation of these tissues. To discern the *in vivo* functions of *LncRNA-HIT* during development will be more challenging, as targeted disruptions of *LncRNA-HIT* should affect 5’ HoxA gene expression either by disrupting activation mediated by the enhancer RNA functions of *LncRNA-HIT* -p100/CBP complexes or by disrupting *cis*-regulatory elements required for 5’ HoxA gene expression. By either mechanism 5’ HoxA gene expression would be reduced, creating phenotypes that cannot be solely attributed to the loss of *LncRNA-HIT* function.

*LncRNA-HIT* is also capable of functioning as a guide RNA as it can recruit the same proteins to loci independent of its site of transcription. By this process *LncRNA-HIT*-p100/CBP complexes can regulate a larger cohort of genes. As an epigenetic modulator of chondrogenesis, *LncRNA-HIT*, may be one of several non-coding RNA species required for the formation and/or maintenance of cartilage tissues including micro RNAs MiR-145 and 337 as well as the small nucleolar RNAs U38 and U48 which are elevated in the serum of individuals affected by injury-induced osteoarthritis [98–99]. These studies in conjunction with the present work indicates a higher level of gene regulation is necessary for the initial formation of cartilage tissues as well as its loss during osteoarthritis, providing additional targets to exploit as therapies to minimize joint tissue loss as a consequence of injury or disease.

## Materials and Methods

### Ethics statement

All investigations using mice were certified as compliant with AVMA guidelines by the OHSU IACUC board prior to implementation following an approved mouse use protocol IS00001648 to HSS. Euthanasia was conducted using CO2 gas until all signs of movement and respiration ceased, followed by prompt thoracotomy. This method is consistent with the recommendations of the Panel on Euthanasia of the American Veterinary Medical Association. PHS 398/2590 (Rev. 06/09).

### Mouse tissues and gestational timing

Embryonic tissues were derived from timed matings of wild type Swiss Webster mice (Charles River Labs). Gestational development was measured in embryonic days (E) where E = 0.5 reflects the detected day of a vaginal plug.

### RNA FISH

Limb bud mesenchyme was dissected from E 11.0 embryos, dissociated, and grown in culture in two chamber cover-glass slides (Nunc Lab- TEKII, Thermo Scientific) as described [55]. After 24H the cells were fixed for 10 min using 4% formaldehyde/PBS and prepared for RNA FISH following the protocol described by the Lanctôt laboratory (http://lanctotlab.org/en/protfish_rnafisholigo20mer.html). The *LncRNA-HIT* RNA FISH probe sets were synthesized and labeled with CalFluor Red 610 by Stellaris Biosearch Technologies (S1 Table). Cells were imaged using a Zeiss Axioplan epifluorecence microscope fitted with a 100x 1.4 NA oil objective and a cooled monochrome CCD camera using a 2 second exposure. Detected signals were pseudo-colored red (*LncRNA-HIT*), green (*Gapdh*), and blue (DAPI) using the Zeiss AxioVision digital image processing software (release 4.7.2).

### p100 and CBP RNA Immunoprecipitation (RIP)

Approximately 1mg of E 11.5 limb bud lysates were used to immunoprecipitate p100 and CBP with endogenous *LncRNA-HIT* transcripts following the previously described RIP procedure [5, 68] using antibodies specific for SND1 (p100) (Cat. Ab65078 Abcam, Cambridge, MA) and CBP (Cat. 7389 Cell Signaling, Danvers, MA). After immunoprecipitation of p100, CBP, or IgG, the co-immunoprecipitated RNA was extracted using TRIzol following the manufacturer’s protocol (ThermoFisher). After extraction, the isolated RNA was treated with RQ1 Rnase-Free Dnase (Cat. M6101 Promega, Madison, WI) and evaluated for enrichment of *LncRNA-HIT* using qRTPCR as described in the RNA isolation and qRTPCR section of the Methods. Three independent immunoprecipitation assays were performed for p100, CBP, and the IgG control analysis of *LncRNA-HIT* co-immunoprecipitation.

### Micromass tissue culture

Embryos were collected at E 10.5 as described [55]. Cells were transfected with two small interfering RNAs (siRNAs) specific for *LncRNA-HIT* or with a scrambled siRNA control at a concentration of 10 nM using Trifectin as recommended (IDT, Coralville, Iowa). 24H after transfection, the cells were trypsinized and resuspended to a final concentration of 2x10^7^ cells/ml and seeded as 10 ul drops into 60 mm Falcon tissue cultures as described [56]. The *LncRNA-HIT* siRNA sequences used were:

1. Antisense 5’ UUAAGGUCACAGACCACCUUGGAGGGU 3’
2. Antisense 5’ CCUCUCUCUCCCUCCCUCCUUUCCUUU 3’

For *Snd1* repression, limb mesenchyme was transfected using a commercial *Snd1* siRNA cocktail at 30 nM concentrations as recommended by the manufacturer (Life Technologies: Cat: 4390771, Grand Island, NY).

### ChIRP-seq analysis

25 oligonucleotide probes corresponding to the murine *LncRNA-HIT* transcript were selected using the Stellaris Probe Designer Software set to masking level 5 (biosearchtech.com) (S2 Table). The oligonucleotide sequences were synthesized with an 18-atom spacer followed by a biotin tag located at the 3’ end and purified by HPLC as described by the manufacturer (IDT, Coralville, Iowa). The autopod regions of the E 11 limbs were dissected and digested for 10 minutes in sterile PBS containing 0.1% trypsin and 0.1% collagenase as described [56]. Approximately 2000 embryonic limb buds were required to produce the 200mg cells needed for each ChIRP assay. Cell lysis and chromatin shearing and streptavidin bead precipitation were accomplished using a Bioruptor instrument (Diagenode) as previously described [50]. As a control, chromatin from murine glial cells (a kind gift from Dr. Peter Hurlin), that do not express *LncRNA-HIT*, were used in parallel assays and hybridized with the same *LncRNA-HIT* probes. The precipitated DNA was quantified using a Qubit 2.0 DNA fluorometer (Life Technologies) and submitted to Elim Biopharm (Hayward, CA) for library preparation and next generation sequencing using an Ilumina HiSeq 2500 sequencer.

### Data analysis

The *LncRNA-HIT* ChIRP-seq libraries were mapped, normalized, and analyzed using the ChIRP-seq analysis pipeline previously described [51]. Raw reads from each ChIRP-seq library were uniquely mapped to the reference mouse genome (mm9) using bowtie-0.12.9 and normalized to total read count as described [52–53]. Even and odd *LncRNA-HIT* ChIRP samples were merged by taking the minimum value of the even and odd tracks at each genomic position. The mouse glial cell line that does not express *LncRNA-HIT* was used as a control to identify false hybridization peaks detected by the *LncRNA-HIT-*specific even and odd probe pools. Peaks were called by MACS using the *LncRNA-HIT* ChIRP merge sample and the DNA input, with an initial threshold p-value ≤ 5.9 x 10^−57^ corresponding to 775 peaks based on visual inspection of the peaks using the UCSC Genome Browser. Peaks were additionally filtered by visual inspection with UCSC Genome Browser excluding any peak with a corresponding peak in the glial controls. The limb-specific peaks were then submitted for analysis using the Genomic Regions of Enrichment of Annotations Tool (GREAT) [54]. Following GREAT the peaks were additionally filtered to identify high confidence peaks mapping within 25 kb of a known gene resulting in the identification of 42 *LncRNA-HIT*-associated genes by visualization using the UCSC Genome Browser. The *LncRNA-HIT* ChIRP-seq data sets have been submitted to the NCBI GEO repository under the accession number, GSE70986.

### Peak validation

42 near-peak genes were validated for regulation by *LncRNA-HIT* -p100/CBP using siRNA-mediated reduction of the native *LncRNA-HIT* transcript followed by gene-specific qRTPCR in limb mesenchyme primary cultures. For each gene, a minimum of three independent *LncRNA-HIT* siRNA treatments followed by qRTPCR was performed. A scrambled siRNA control was used in parallel assays for each validation experiment. Genes exhibiting decreased expression ≤ -1.5-fold, were assessed for their collective ontological function using the GO consortium ontology tool kit, AmiGo version 2.1.4, using the criteria: experimental biological processes in *M*. *musculus* (http://amigo2.berkeleybop.org/amigo) [100].

### RNA Isolation and qRTPCR

RNA was isolated using TRIzol (Life Technologies, Grand Island, NY) as instructed by the manufacturer. Gene expression levels were quantitated using a real-time quantitative reverse transcriptase polymerase chain reaction method (qRTPCR). First-strand cDNA was synthesized using an ImProm-II Reverse Transcription System (Promega, Madison, WI). A minimum of three independent samples were used for qRTPCR using a SYBR Green PCR Super Mix and an IQ5 thermal cycler according to the manufacturer’s instructions (BioRad Hercules, CA). Fold change expression levels were determined after normalization of the amplification products to *Gapdh* expression using the BioRad IQ5 software suite. Student’s t-tests were used to determine statistical significance. Data was plotted using Sigmaplot 10.0 (Systat, San Jose, CA). Primer sequences used for the qRTPCR assays are presented in S3 Table.

### Alcian blue staining

Cartilage nodule formation in the siRNA-treated micromass cultures was visualized by staining the micromass cultures with Alcian blue 8GX (Canemeo Inc, QC, Canada), as described [101]. Alcian blue stained nodules were photographed using a Leica MZFLIII stereoscope fitted with a Canon EOS 40D digital camera.

### RNA tethering assay

RNA tethering assays were performed using expression vectors encoding GAL4-p100 andGAL4-λN as described [5, 64]. Expression plasmids containing five copies of the *BoxB* transcript or containing the same five copies of the *BoxB* transcript fused to the *LncRNA-HIT* transcript (*BoxB-LncRNA-HIT*) were subsequently transfected into NG108-15 cells with the GAL4-λN and/or the GAL4-p100 expression vectors using increasing dosages of the *BoxB* or *BoxB-LncRNA-HIT* vectors at 0, 50, 100, or 250 ng per assay and assessed for luciferase expression 48H after transfection using the Dual-Luciferase Reporter Assay System (Promega, Madison, WI). All analyses were performed in triplicate and the average and standard deviation were plotted using Sigmaplot 10.1.

### *LncRNA-HIT* WDR5 interaction analysis

Flag-WDR5 293T cells were transfected with a *LncRNA-HIT* expression vector and evaluated for WDR5-*LncRNA-HIT* interactions by RNA protein co-immunoprecipitation as described [102].

### RNA affinity assay

Mouse *LncRNA-HIT* and *U1* genes were cloned into the pCDNA3 vector (Life Technologies, Grand Island, NY) and transcribed using T7 RNA polymerase and biotin-16-UTP as described by the manufacturer (Roche, Indianapolis, IN). Cell lysates from E11.0 wild type embryos limbs which were homogenized in mRIPA buffer and centrifuged as described [103]. 1 mg of total protein was incubated with either the *LncRNA-HIT* or *U1* transcripts tagged with biotin-16-UTP 12 H at 4°C. Following incubation, the RNA-protein mixtures were incubated with 100 μl prewashed streptavidin beads for 2.5 H at 4°C. After incubation, the streptavidin beads were washed five times in ice cold PBS and combined with 50 μl of an SDS elution buffer containing 125 mM Tris/HCl pH 6.8, 20% glycerol, 4% SDS, 10% 2-mercaptoethanol and 0.02% bromphenol blue. After two rounds of elution, the recovered protein mixture was heated to 95°C for 5 minutes and fractionated by SDS-PAGE electrophoresis using a 4–12% gradient gel (Nu Page, Invitrogen). The gels were stained with Coomassie blue and the individual bands digested with trypsin and submitted for identification by mass spectroscopy by the OHSU Proteomics Core Facility as described [104].

### *In situ* hybridization

The antisense riboprobe specific for mouse *LncRNA-HIT* was generated using PCR amplification of the gene-specific sequence. The amplifying primers were: 5’-AGAGGAGGTTCCCAGACTCC-3’, and 5’-GCACACAAACACTGATATGCAA-3’. Riboprobe synthesis and *in situ* hybridization was performed on mouse embryos as described [105].

### Quantitative Chromatin immunoprecipitation (qChIP)

Limb buds from E 11.0 embryos were dissected, and the mesenchymal cells were transfected with the previously described *LncRNA-HIT* siRNAs. 24 H after transfection, qChIP assays were performed as described [106–108], using an anti-acetyl histone H3 (lys27) antibody (Cat. 07–360, Millipore, Billerica, MA) or mouse IgG (Diagenode: C15400001) as a negative control. DNA sequences specific to H3K27ac peaks proximal to *LncRNA-HIT* -associated peaks were identified using an overlay of the E 10.5 limb bud H3K27ac ChIP-seq dataset [63] and the UCSC Genome Browser. Primers were selected to amplify the DNA regions containing the H3K27ac *peaks* using Primer3 (simgene.com) (S4 Table). ChIP-enriched H3K27ac fragments were quantified by qPCR using an IQ5 quantitative PCR instrument (BioRad, Hercules, CA) as described [107–108].

## Supporting Information

S1 Fig *LncRNA-HIT* gene sequence, conservation and translational analysis. **(A)** Location of *LncRNA-HIT*, *Hoxa11*, *Hoxa13*, and *Hottip* on mouse Chromosome 6. Numerical values represent predicted physical distances in kilobase pairs. Arrows represent the direction of gene transcription. **(B)** Sequence of *9530018H14RIK*. Conserved region is underlined in red. Sequences underlined in black represent the potential RNA nuclear retention signal. **(C)** Translation of the *LncRNA-HIT* transcript in all six reading frames. Pink Bars represent stop codons in each reading frame. Green Bars represent initiation codons present in each reading frame. **(D**). *LncRNA-HIT* (9530018H14Rik) probe sets included on the Affymetrix MOE 430 2.0 Mouse gene chip. **(E)** Conservation of the sequence in multiple vertebrate species.
(TIF)

S2 Fig Assessment of *LncRNA-HIT*, *Hoxa13*, and *Hottip* for protein coding potential. **(A)** Coding potential assessment using CPAT which determined low coding potential for *LncRNA-HIT* and *Hottip*, and high coding potential for *Hoxa13*. **(B)** Initiation codon translational analysis using NetStart for *LncRNA-HIT*, *Hoxa13*, and *Hottip*. Stop codons are depicted with the letter N, favorable initiation codons are depicted with the letter i.
(TIF)

S3 Fig RIP qRTPCR analysis of *LncRNA-HIT*, *Hotair*, or *U1* RNA binding to WDR5. Blue bars represent gene-specific enrichment after precipitation of FLAG-tagged WDR5 compared to an IgG precipitated control (pink bars). Note that the precipitation of WDR5 did not produce an enrichment of the *LncRNA-HIT* transcript.
(TIF)

S4 Fig *LncRNA-HIT* and U1 RNA affinity chromatography identifies a specific interaction between *LncRNA-HIT* and p100 in E 11.0 limb bud cell lysates. Proteins bound to the *LncRNA-HIT* RNA were eluted using increasing molar amounts of NaCl or Guanidine and fractionated using PAGE. Individual bands were sequences by mass spectroscopy. A prominent 100 Kd band (asterisk) eluting from the *LncRNA-HIT* column but not the U1 column was identified as p100 by protein mass spectrometry.
(TIF)

S5 Fig GREAT analysis of *LncRNA-HIT* peak distribution in the murine genome. **(A)** Genomic regions with associated gene regulatory regions. Red bar represent the number of *LncRNA-HIT* peaks associating with genomic regions lacking a known *cis*-regulatory function. Open bars represent the number of *LncRNA-HIT* peaks associating with previously characterized *cis*-regulatory element capable of regulating one or more genes. **(B).** Distance between the *LncRNA-HIT* associated genomic regions known translational start sites (TSS). **(C)** Absolute physical distance between *LncRNA-HIT* associated peaks and TSS sites. **(D)** GO analyses based on the association of the lncRNA with known cis-regulatory elements. Note that no functional terms were detected for the *LncRNA-HIT* peaks, suggesting the lncRNA may associate with novel cis-regulatory elements or may function at non *cis* regulatory regions to regulate near-peak gene expression.
(TIF)

S6 Fig Ontological analysis of the *LncRNA-HIT*-regulated genes using AMIGO 2.0 for experimental biological processes. Note that significant ontologies were detected for proximal distal pattern formation and the regulation of chondrocyte differentiation.
(TIF)

S1 Table Nucleotide sequences for the LncRNA-HIT RNA FISH probe set. (DOCX)

S2 Table LncRNA-HIT ChIRP probe set. (DOCX)

S3 Table qRTPCR primers used to validate LncRNA-HIT regulation of peak-associated genes. (DOCX)

S4 Table qPCR primers used to detect changes in H3K27ac fragment enrichment. (DOCX)

S5 Table Sequences associated with murine H3K27ac peaks proximal to the promoter regions of *Skap*2 and *Creb5*. (DOCX)
