# Supplementary figures and images for: LncRNA-HIT Functions as an Epigenetic Regulator of Chondrogenesis through Its Recruitment of p100/CBP Complexes
Source: PLoS Genet. 2015 Dec 3;11(12):e1005680. doi: 10.1371/journal.pgen.1005680 (PMC4669167; doi:10.1371/journal.pgen.1005680)

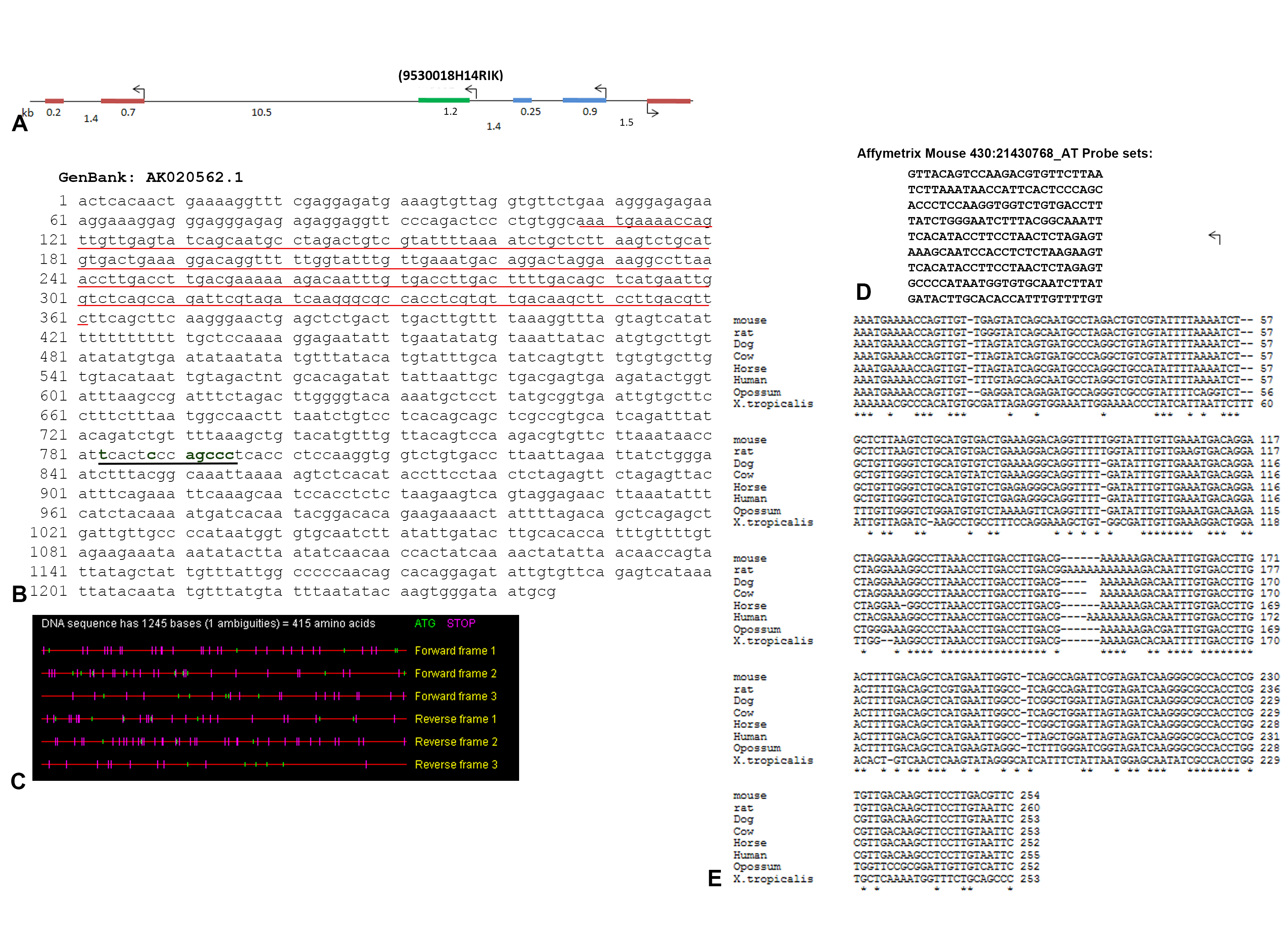

Supplement: S1 Fig — (A) Location of LncRNA-HIT, Hoxa11, Hoxa13, and Hottip on mouse Chromosome 6. Numerical values represent predicted physical distances in kilobase pairs. Arrows represent the direction of gene transcription. (B) Sequence of 9530018H14RIK. Conserved region is underlined in red. Sequences underlined in black represent the potential RNA nuclear retention signal. (C) Translation of the LncRNA-HIT transcript in all six reading frames. Pink Bars represent stop codons in each reading frame. Green Bars represent initiation codons present in each reading frame. (D). LncRNA-HIT (9530018H14Rik) probe sets included on the Affymetrix MOE 430 2.0 Mouse gene chip. (E) Conservation of the sequence in multiple vertebrate species. (TIF) [file pgen.1005680.s001.tif]

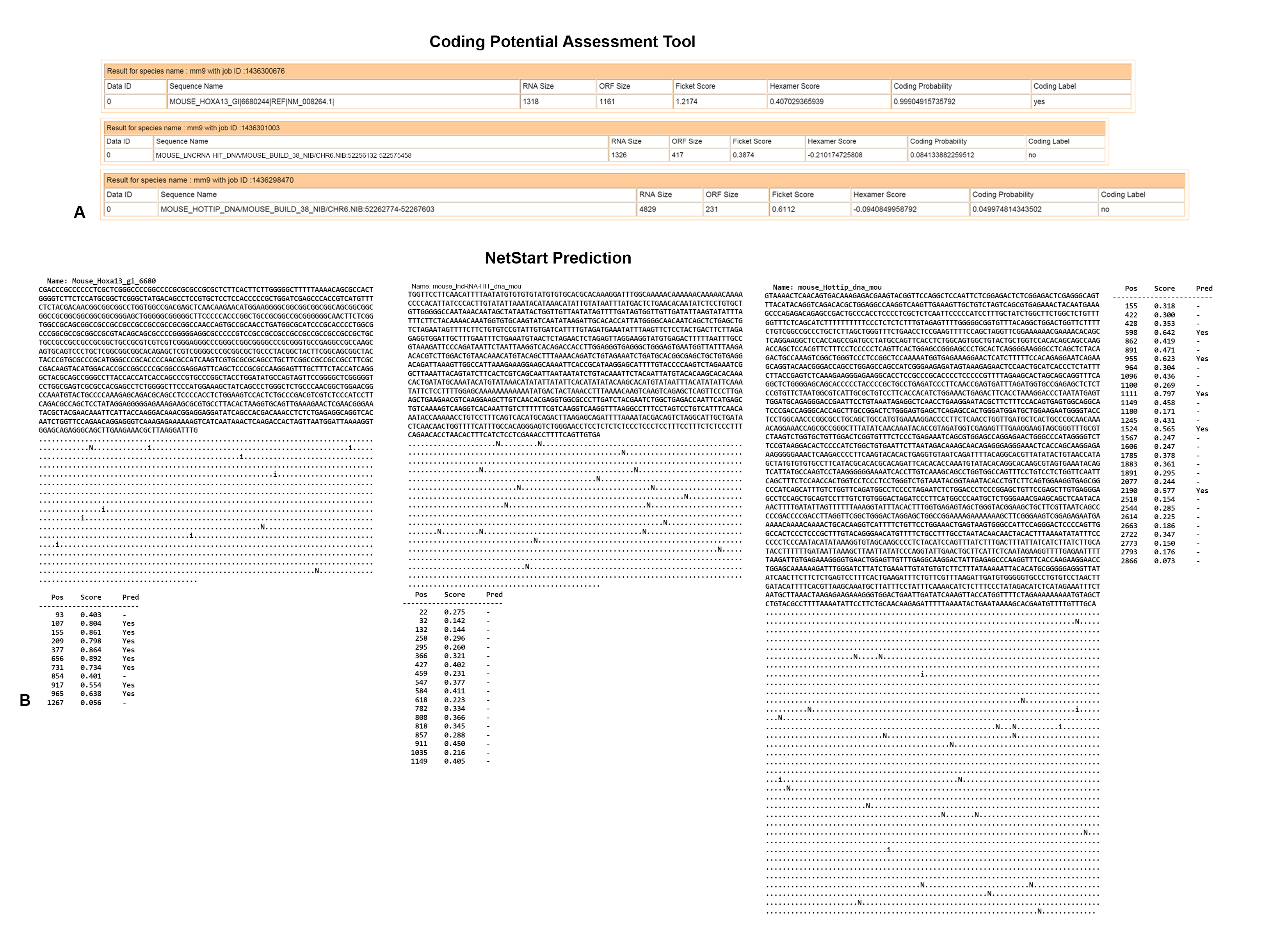

Supplement: S2 Fig — (A) Coding potential assessment using CPAT which determined low coding potential for LncRNA-HIT and Hottip, and high coding potential for Hoxa13. (B) Initiation codon translational analysis using NetStart for LncRNA-HIT, Hoxa13, and Hottip. Stop codons are depicted with the letter N, favorable initiation codons are depicted with the letter i. (TIF) [file pgen.1005680.s002.tif]

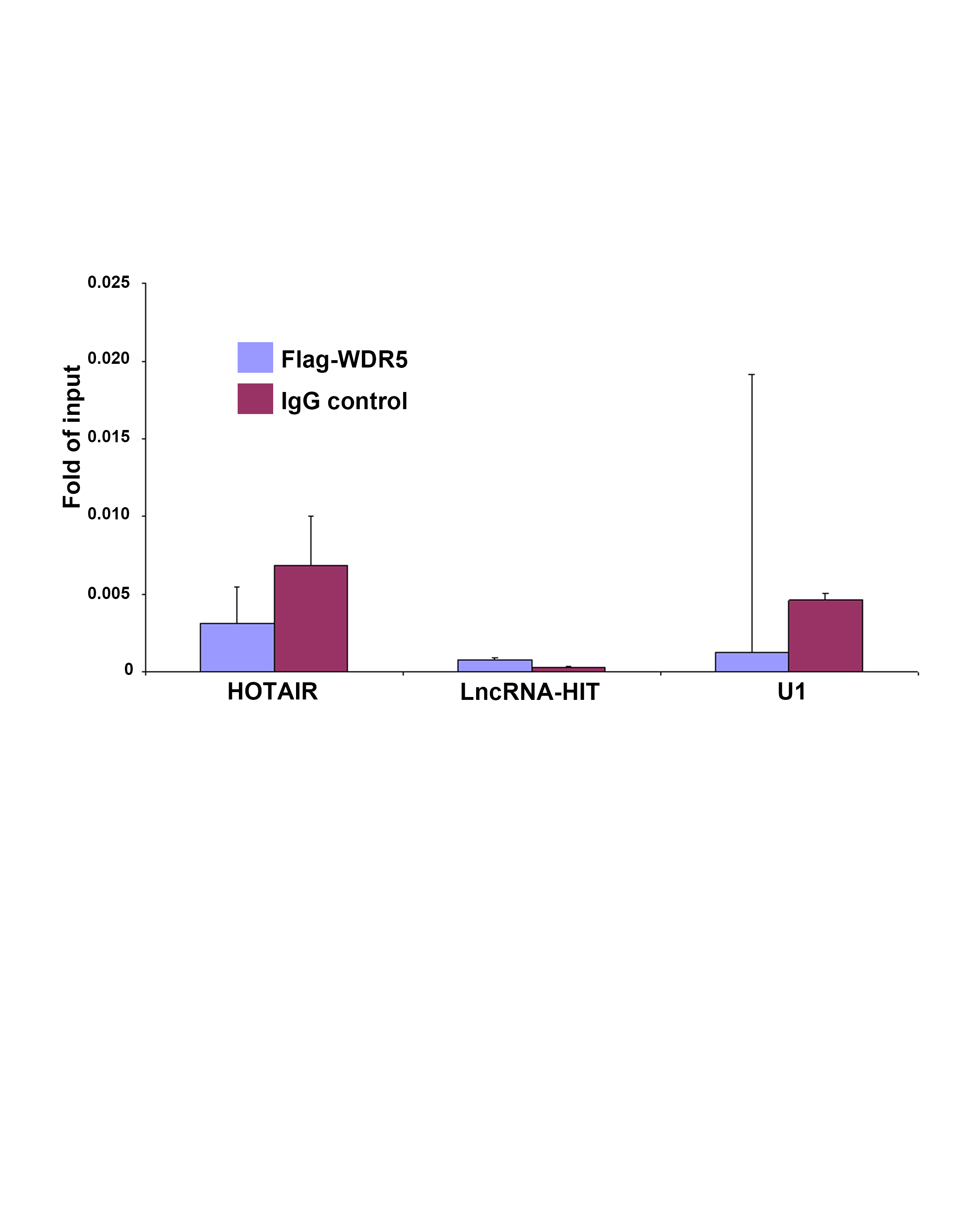

Supplement: S3 Fig — Blue bars represent gene-specific enrichment after precipitation of FLAG-tagged WDR5 compared to an IgG precipitated control (pink bars). Note that the precipitation of WDR5 did not produce an enrichment of the LncRNA-HIT transcript. (TIF) [file pgen.1005680.s003.tif]

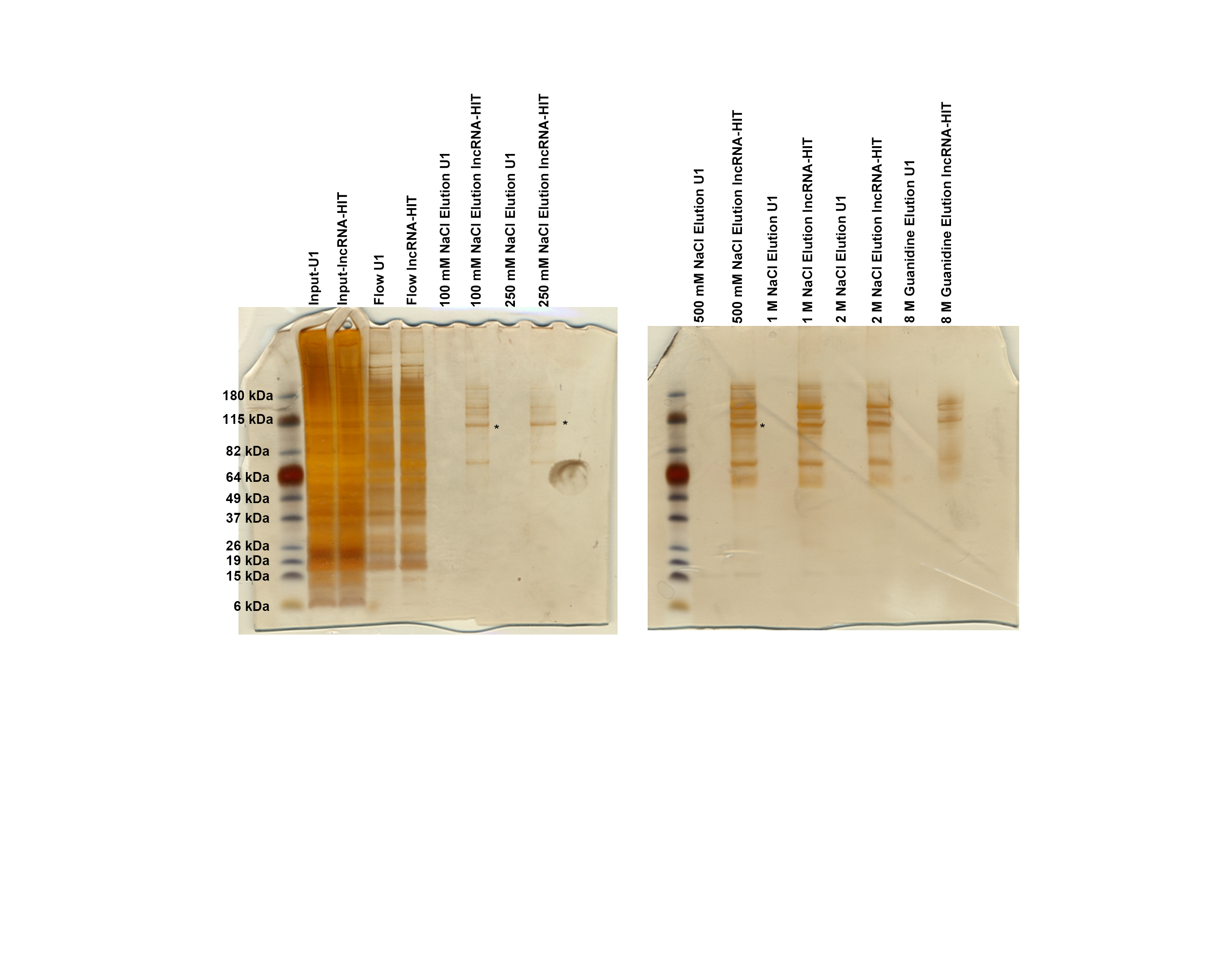

Supplement: S4 Fig — Proteins bound to the LncRNA-HIT RNA were eluted using increasing molar amounts of NaCl or Guanidine and fractionated using PAGE. Individual bands were sequences by mass spectroscopy. A prominent 100 Kd band (asterisk) eluting from the LncRNA-HIT column but not the U1 column was identified as p100 by protein mass spectrometry. (TIF) [file pgen.1005680.s004.tif]

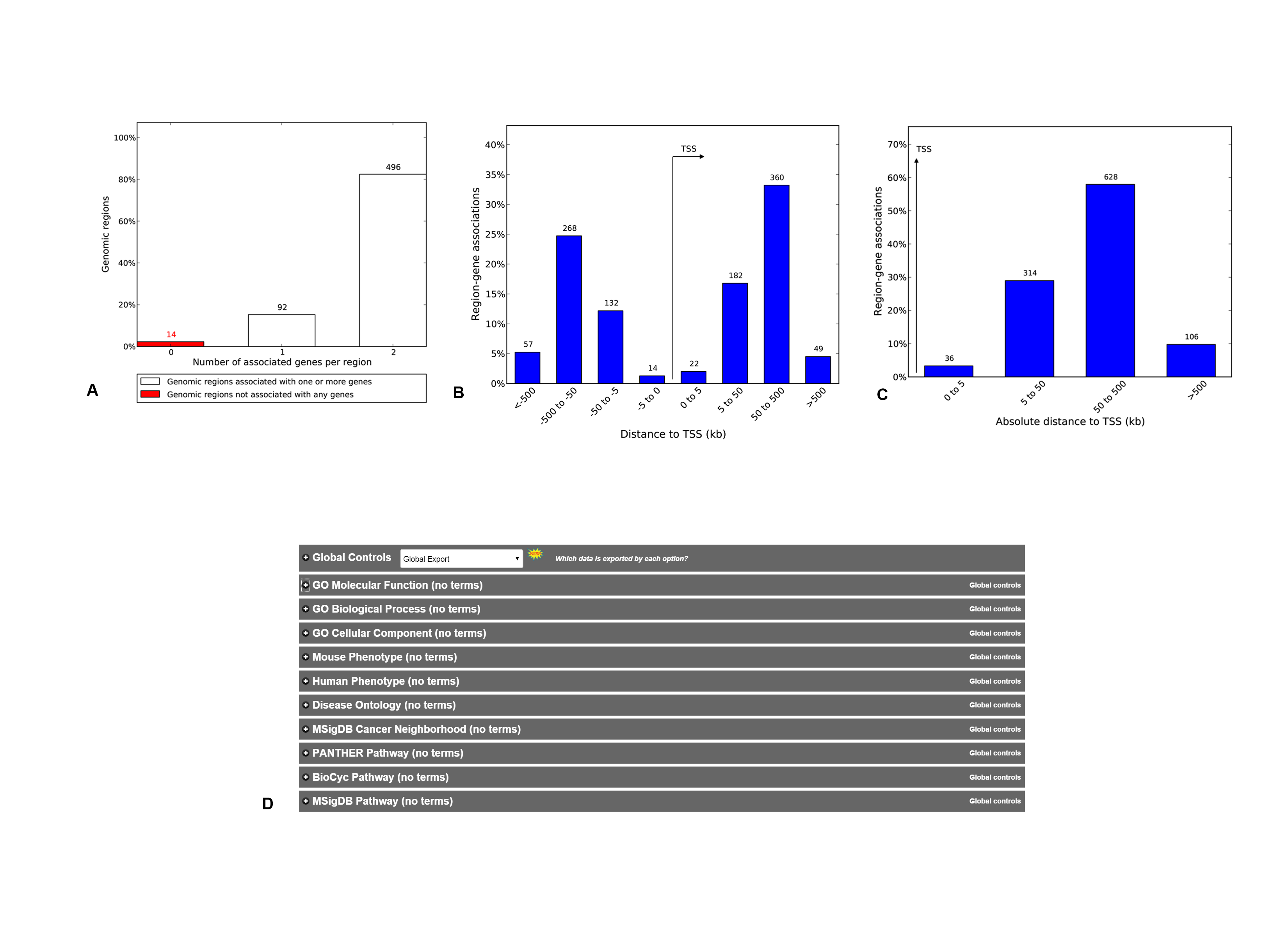

Supplement: S5 Fig — (A) Genomic regions with associated gene regulatory regions. Red bar represent the number of LncRNA-HIT peaks associating with genomic regions lacking a known cis-regulatory function. Open bars represent the number of LncRNA-HIT peaks associating with previously characterized cis-regulatory element capable of regulating one or more genes. (B). Distance between the LncRNA-HIT associated genomic regions known translational start sites (TSS). (C) Absolute physical distance between LncRNA-HIT associated peaks and TSS sites. (D) GO analyses based on the association of the lncRNA with known cis-regulatory elements. Note that no functional terms were detected for the LncRNA-HIT peaks, suggesting the lncRNA may associate with novel cis-regulatory elements or may function at non cis regulatory regions to regulate near-peak gene expression. (TIF) [file pgen.1005680.s005.tif]

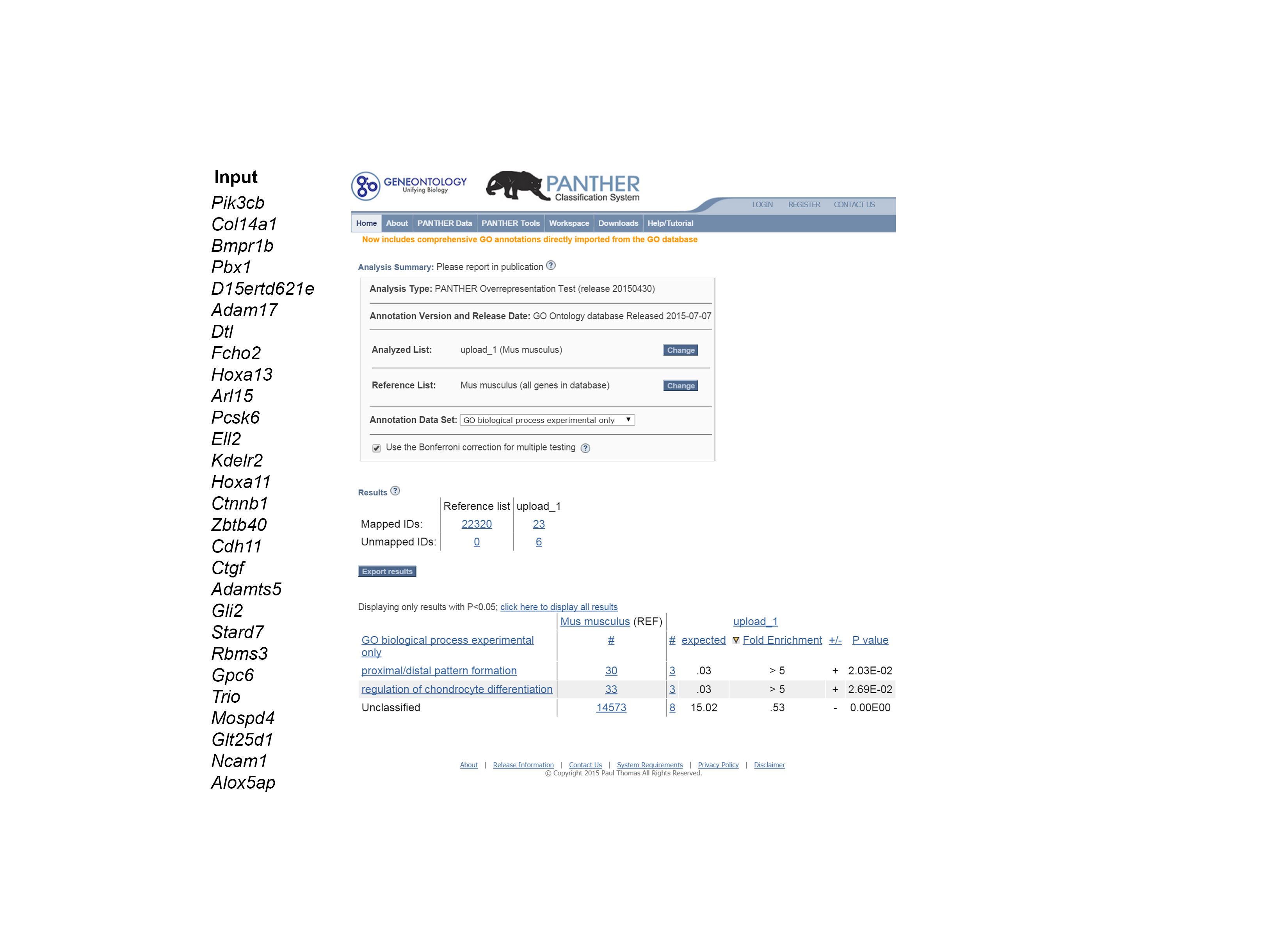

Supplement: S6 Fig — Note that significant ontologies were detected for proximal distal pattern formation and the regulation of chondrocyte differentiation. (TIF) [file pgen.1005680.s006.tif]
