# Supplementary material for: LncRNA-HIT Functions as an Epigenetic Regulator of Chondrogenesis through Its Recruitment of p100/CBP Complexes
Source: PLoS Genet. 2015 Dec 3;11(12):e1005680. doi: 10.1371/journal.pgen.1005680 (PMC4669167; doi:10.1371/journal.pgen.1005680)
Supplement: S1 Table — (DOCX) [file pgen.1005680.s007.docx]

| \| S1 Table LncRNA-HIT RNA FISH probe set \| \| \| --- \| --- \| \| Probe \| 5'-3' Sequence \| \| 1 \| CATTTGCCACAGGGAGTCTG \| \| 2 \| CTGATACTCAACAACTGGTT \| \| 3 \| catttgccacagggagtctg \| \| 4 \| ctgatactcaacaactggtt \| \| 5 \| aaatacgacagtctaggcat \| \| 6 \| cacatgcagacttaagagca \| \| 7 \| accaaaaacctgtcctttca \| \| 8 \| ctagtcctgtcatttcaaca \| \| 9 \| caaggtcaaggtttaaggcc \| \| 10 \| gtcaaggtcacaaattgtct \| \| 11 \| gaccaattcatgagctgtca \| \| 12 \| cttgatctacgaatctggct \| \| 13 \| gaagcttgtcaacacgaggt \| \| 14 \| tcactcgtcagcaattaata \| \| 15 \| atcggcttaaataccagtat \| \| 16 \| attttgtaccccaagtctag \| \| 17 \| cacaattcaccgcataagga \| \| 18 \| ttaagaacacgtcttggact \| \| 19 \| gtgagggctgggagtgaatg \| \| 20 \| attaaggtcacagaccacct \| \| 21 \| gagactttttaatttgccgt \| \| 22 \| actctagagttaggaaggta \| \| 23 \| gtccgtattgtgatcatttt \| \| 24 \| aacaatcagctctgagctgt \| \| 25 \| agattgcacaccattatggg \| \| 26 \| caaatggtgtgcaagtatca \| \| 27 \| gttttgatagtggttgttga \| \| 28 \| gggggccaataaacaatagc \| \| 29 \| acacaatatctcctgtgctg \| |
| --- | --- | --- | --- | --- | --- | --- | --- | --- | --- | --- | --- | --- | --- | --- | --- | --- | --- | --- | --- | --- | --- | --- | --- | --- | --- | --- | --- | --- | --- | --- | --- | --- | --- | --- | --- | --- | --- | --- | --- | --- | --- | --- | --- | --- | --- | --- | --- | --- | --- | --- | --- | --- | --- | --- | --- | --- | --- | --- | --- | --- | --- | --- |
