# Supplementary material for: LncRNA-HIT Functions as an Epigenetic Regulator of Chondrogenesis through Its Recruitment of p100/CBP Complexes
Source: PLoS Genet. 2015 Dec 3;11(12):e1005680. doi: 10.1371/journal.pgen.1005680 (PMC4669167; doi:10.1371/journal.pgen.1005680)
Supplement: S2 Table — (DOCX) [file pgen.1005680.s008.docx]

| S2 Table. LncRNA-HIT ChIRP-seq probe sets  Probe  1 5’-CAT TTG CCA CAG GGA GTC TG/iSp18//3Bio/-3’  2 5’-CTG ATA CTC AAC AAC TGG TT/iSp18//3Bio/-3’  3 5’-AAA TAC GAC AGT CTA GGC AT/iSp18//3Bio/-3’  4 5’-CAC ATG CAG ACT TAA GAG CA/iSp18//3Bio/-3’  5 5’-ACC AAA AAC CTG TCC TTT CA/iSp18//3Bio/-3’  6 5’-CTA GTC CTG TCA TTT CAA CA/iSp18//3Bio/-3’  7 5’-CAA GGT CAA GGT TTA AGG CC/iSp18//3Bio/-3’  8 5’-GTC AAG GTC ACA AAT TGT CT/iSp18//3Bio/-3’  9 5’-GAC CAA TTC ATG AGC TGT CA/iSp18//3Bio/-3’  10 5’-CTT GAT CTA CGA ATC TGG CT/iSp18//3Bio/-3’  11 5’-GAA GCT TGT CAA CAC GAG GT/iSp18//3Bio/-3’  12 5’-ATC TTC ACT CGT CAG CAA TT/iSp18//3Bio/-3’  13 5’-CCA AGT CTA GAA ATC GGC TT/iSp18//3Bio/-3’  14 5’-GAA ATC TGA TGC ACG GCG AG/iSp18//3Bio/-3’  15 5’-TTA AGA ACA CGT CTT GGA CT/iSp18//3Bio/-3’  16 5’-GTG AGG GCT GGG AGT GAA TG/iSp18//3Bio/-3’  17 5’-ATT AAG GTC ACA GAC CAC CT/iSp18//3Bio/-3’  18 5’-GAG ACT TTT TAA TTT GCC GT/iSp18//3Bio/-3’  19 5’-ACT CTA GAG TTA GGA AGG TA/iSp18//3Bio/-3’  20 5’-GTC CGT ATT GTG ATC ATT TT/iSp18//3Bio/-3’  21 5’-AGA TTG CAC ACC ATT ATG GG/iSp18//3Bio/-3’  22 5’-CAA ATG GTG TGC AAG TAT CA/iSp18//3Bio/-3’  23 5’-GTT TTG ATA GTG GTT GTT GA/iSp18//3Bio/-3’  24 5’-GGG GGC CAA TAA ACA ATA GC/iSp18//3Bio/-3’  25 5’-ACA CAA TAT CTC CTG TGC TG/iSp18//3Bio/-3’ |
| --- |
