# Supplementary material for: LncRNA-HIT Functions as an Epigenetic Regulator of Chondrogenesis through Its Recruitment of p100/CBP Complexes
Source: PLoS Genet. 2015 Dec 3;11(12):e1005680. doi: 10.1371/journal.pgen.1005680 (PMC4669167; doi:10.1371/journal.pgen.1005680)
Supplement: S3 Table — (DOCX) [file pgen.1005680.s009.docx]

| **S3 Table qRTPCR primers used to validate LncRNA-*HIT*-regulated loci**  Pik3cbF 5'-CCATATCCGTCGATTTCCTT-3'  Pik3cbR 5'-CGAGTCAATGTCCATGAGGA-3'  LncRAN-HITF 5'- CCAGACTCCCTGTGGCAAATGAAA-3'  LncRNA-HITR 5'-CATGAGCTGTCAAAAGTCAAGGTCAC-3'  Col14a1F 5'-TGCTTGTTTCTGCACCATTG-3'  Col14a1R 5'-AGCCACCAAATTTCCCTCTT-3'  Bmpr1bf 5'-GGCCCAAGATCCTACGTTGT-3'  Bmpr1bR 5'-CATCCAGAGGTGACAACAGG-3'  Pbx1F 5'-CAGGACATCGGGGACATTT-3'  Pbx1R 5'-CACATTAAACAAGGCAGGCTTC-3'  D15ertd621eF 5'-CATCGACGTTGAGTTCCACA-3'  D15ertd621eR 5'-CGCAACTGATTGCGAATACT-3'  Adam17F 5'-CTGACCACTTTGGTGCCTTT-3'  Adam17R 5'-TCCTTATGGAGTGCTGCTGA-3'  DtlF 5'-GCCCAGATGCTCTTCAACTC-3'  DtlR 5'-TGGGACTCCTGTTTCTCCAT-3'  Fcho2F 5'-CATTTCGTGGAGAATTTCTGG-3'  Fcho2R 5'-TCATTGATCTGGAGTATGCCTCT-3'  HoxA13F 5'-CCAAATGTACTGCCCCAAAGAGC-3'  Hoxa13R 5'-CGTAGCGTATTCCCGTTCGAGTT-3'  Arl15F 5'-TGAGGCGTTTCTGTACATGG-3'  Arl15R 5'-GACAGCAGGCTGGTTTTCC-3'  Pcsk6F 5'-CTACCTCAACTTGGGCCAGA-3'  Pcsk6R 5'-GGGTCCATTCTGAGGAAGGT-3'  Ell2F 5'-ACAACATCACCGTGCTGCAT-3'  Ell2R 5'-CCTTGGAGTCCTTGGAACTG-3'  Kdelr2F 5'-GCCATCGTCATCCTACTGCT-3'  Kdelr2R 5'-TGAAGGAAGTGAAAAGGTCCA-3'  Hoxa11F 5'-GTCTTCCGGCCACACTGA-3'  Hoxa11R 5'-GACAGTTGCAG CGCTTCTCT-3'  Ctnnb1F 5'-AAGGGCAACCCTGAGGAAGA-3'  Ctnnb1R 5'-CATTGCATACTGCCCGTCAA-3'  Zbtb40F 5'-CCTGTTGGACAAATCCCAAG-3'  Zbtb40R 5'-GCCAGTCTTCTCAGGGTGTC-3'  Cdh11F 5'-CTGCAACGCTCCAAGAGAG-3'  Cdh11R 5'-ATGTTCCCATCACCGGAGT-3'  CtgfF 5'-TGTGCACTGCCAAAGATGGT-3'  CtgfR 5'-TCCAGGCAAGTGCATTGGTA-3'  Adamts5F 5'-TGGCAGCACCA CATAACCA-3'  Adamts5R 5'-CCCAGGGTGTCACATGAATGA-3'  Gli2F 5'-AGCCCAGCCTTCACTTTTCC-3'  Gli2R 5'-AGGGGTGGTGTGTGTCCAAA-3'  Stard7F 5'-GGCCATGCCTCTGTTTTG-3'  Stard7R 5'-TGACATTTCTTCCAACCGTTTC-3'  Pcsk6F 5'-CTACCTCAACTTGGGCCAGA-3'  Pcsk6R 5'-GGGTCCATTCTGAGGAAGGT-3'  Rbms3F 5'-AAAGCAGTGGCGTCCTTGAA-3'  Rbms3R 5'-TCCAGCTCCTGCTCATCCAT-3'  Gpc6F 5'-CTTACGGTGCCAAGGGATT-3'  Gpc6R 5'-CTGTTGACTCAGCTTGTCTTCC-3'  TrioF 5'-CCAAGGACCTAGCCGACAT-3'  TrioR 5'-AATGGGACCTCCACGCTTAT-3'  Mospd4F 5'-AGCACAAGTTCATGGTGCAG-3'  Mospd4R 5'-CGGTTCAAACACACACCTCA-3'  Glt25d1F 5'-TCGTCTTTGCCTTCTCCTGT-3'  Glt25d1R 5'-AAGCTCTCTGCTTCGTCCTG-3'  Ncam1F 5'-AAGTGTGTGGTCACGGCTGA-3'  Ncam1R 5'-ACAGCATCCTCCCCTTCCTT-3'  Alox5apF 5'-CCTTGTCACCCTCATCAGC-3'  Alox5apR 5'-AGTGTAGACCCGCTCAAAGG-3'  Ralgps2F 5'-GGCAGGCAAGCAGTGTTACT-3'  Ralgps2R 5'-CGCGTATTCTTCTGGCGTA-3'  Col5a3F 5'-ACAGGGTGACCGAGCATTCA-3'  Col5a3R 5'-TCAGTGTGAGCAGCCAGAAAA-3'  Acss3F 5'-TACAAGCCCTGGACCAAAAC-3'  Acss3R 5'-CTTATCCCCTTGGCCATTTT-3'  Akap6F 5'-CAGACAGCAAGCATGTGGAT-3'  Akap6R 5'-ACAGCAGCTTCAGGGAGAAC-3'  Lynx1F 5'-CCATCTGCTCACAGTGTTCC-3'  Lynx1R 5'-TTCGTGTGGTCATACAGTAGGTG-3'  Sap130F 5'-TTCCGCAGGACTGATAAACC-3'  Sap130R 5'-TAGGGCCTTACCACAACTGG-3'  Pde11aF 5'-CCCTGCAGTAGCACAGAGAA-3'  Pde11aR 5'-AATCTTCGGTCCTGGTAGGC-3'  Vwa5b1F 5'-TGACAGCCTCCCTCACCTAC-3'  Vwa5b1R 5'-CAACACGGTCAGCAATGACT-3'  Tmem132dF 5'-TAGCTGCCCTTTTCTCCAAA-3'  Tmem132dR 5'-AGAAGGAGACATCCGCATTG-3'  Unc13aF 5'-GGTGCCCAAGAGAAGTTCAA-3'  Unc13aR 5'-GCGGTTGATCTCAAACATGA-3'  TpoF 5'-ATGGCAATAATGCTGGTGGT-3'  TpoR 5'-ACCATGAGCTGGCTCGTTT-3'  Mctp1F 5'-AAAGTTTAGCTGCCCGTGAT-3'  Mctp1R 5'-CCTCCCACACAGGATTTAGG-3'  NuggcF 5'-CTCGGAGTAGCACCTCTTGG-3'  NuggcR 5'-CAGAAGGAAAGGCCTGGAAT-3'  Ppp1r32F 5'-ACTCCCTCTTGGGGTTGTCT-3'  Ppp1r32R 5'-TGTAGCCTGTGCCTACATGG-3' |
| --- |
