# Supplementary material for: LncRNA-HIT Functions as an Epigenetic Regulator of Chondrogenesis through Its Recruitment of p100/CBP Complexes
Source: PLoS Genet. 2015 Dec 3;11(12):e1005680. doi: 10.1371/journal.pgen.1005680 (PMC4669167; doi:10.1371/journal.pgen.1005680)
Supplement: S4 Table — (DOCX) [file pgen.1005680.s010.docx]

| **S4 Table qPCR Primers to detect H3K27ac ChIP-fragments at the *HoxA11*-*HoxA13* locus and at the top-five loci impacted by the lncRNA-HIT siRNA treatments**  **Hoxa11-Hoxa13 locus**  Peak 1F 5'-GATAAGTACAGAGTGCTCGATGCTGAG-3'  Peak 1R 5'-CGCCTATGGACCCCCATC-3'  Peak 2F 5'-GCTGTACATGTTTGTTACAGTCCAAGA-3'  Peak 2R 5'-TGTGAGACTTTTTAATTTGCCGTAAAGAT-3'  Peak 3F 5'-GCTGACGAGTGAAGATACTGTAATTTAAGC-3'  Peak 3R 5'-GTTGGCCATTAAAGAAAGGAAGCA-3'  Peak 4F 5'-TGACAAGCTTCCTTGACGTTC-3'  Peak 4R 5'-TTTACATATATTCAAATATTCTCCTTTTGGAGCAA-3'  Peak 5F 5'-TTTGTGACCTTGACTTTTGACAGCTC-3'  Peak 5R 5'-CTGAAGAACGTCAAGGAAGCTTGT-3'  Peak 6F 5'-GGTGTTCTGAAAGGGAGAGAAAGGA-3'  Peak 6R 5'-ACGACAGTCTAGGCATTGCTGA-3'  Peak 7F 5'-TTGTTAAACATTTTGCTAGGGACATCA-3'  Peak 7R 5'-TGCAGACATCTAAATATTTTGGGAGAA-3'  Peak 8F 5'-CCAACACTGTCAAAGGATTTTTCCA-3'  Peak 8R 5'-CCTTCCAGGAGAAGGGGCATA-3'  Peak 9F 5'-CCTGCCATTGTTCCTTCTGGA-3'  Peak 9R 5'-TGGATGAGCGTTCTCTCTCCTC-3'  Peak 10F 5'-TCATCAGCCCCTAAAACACACA-3'  Peak 10R 5'-TTCAGATTACCATCTAACACAGTGTCC-3'  Peak 11F 5'-TGGAACTCTGGGCGATTTCA-3'  Peak 11R 5'-TTGCTCTTTGCCACGAATGC-3'  Peak 12F 5'-GGACTTGGGGCAAGCTGAGA-3'  Peak 12R 5'-ACCCCAATCCGGTCATTCTG-3'  Peak 13F 5'-CCCTCCCAAGCCCTTGACAT-3'  Peak 13R 5'-ATGCTTCTCCAACGAAGCCTTT-3'  Peak 14F 5'-TGTGAGTGAATGGTGCTTTCTTCA-3'  Peak 14R 5'-TGCATACTGCCACATTGTCCA-3'  **Loci not linked to HoxA locus**  Pik3cb-peak F 5'-CACGTCCCCCTGCCCTCT-3'  Pik3cb-peak R 5'-AAAACCATCCTCTGCCAAAAATCA-3'  Col14a1-peak F 5'-CATTCCTCTTAGCAGTTTGGGAGGA-3'  Col14a1-peak R 5'-CAAAAGGAACTTAAAGATAGGCTCTCTGC-3'  Pbx1-peak F 5'-TGCATCCCTTTAATCCGAGAGC-3'  Pbx1-peak R 5'-TCTGCAAATCCCTGGCTGTTC-3'  D15ertd621e-peak F 5'-TCAGCTACGTGGTCCTGTGTGA-3'  D15ertd621e-peak R 5'-TGGTTGAGAGCAGGAAAGAACCA-3'  Bmpr1b-peak1 F 5'-CATGTTAGGCATATTTTTACCAGTTCGTAGA-3'  Bmpr1b-peak1 R 5'-GGCACCAGGTCGTGATAGGG-3'  Bmpr1b-peak 2 F 5'-CTAGCTCATGGAAATTGAAGATGCTTA-3'  Bmpr1b-peak 2 R 5'-GAGAGCCCAGTGCTTCATCCT-3'  **Control loci**  Skap2 Peak1 F 5'-AAACCTTGGTGGAAAAGACCAGT-3'  Skap2 Peak1 R 5'-TGGTATGTCAGCCACACCTCTC-3'  Creb5 Peak1 F 5'-CCTCTGGCTCTGCTGCTCAC-3'  Creb5 Peak1R 5'-CCTGGGGCTGTTTTGGTTACA-3'  Creb5 Peak2 F 5'-CCAGTAAACAGACTAAGAAACAGTGCAG-3'  Creb5 Peak2 R 5'-CCACCCCTGGTCACAGACAC-3 |
| --- |
