# Supplementary material for: LncRNA-HIT Functions as an Epigenetic Regulator of Chondrogenesis through Its Recruitment of p100/CBP Complexes
Source: PLoS Genet. 2015 Dec 3;11(12):e1005680. doi: 10.1371/journal.pgen.1005680 (PMC4669167; doi:10.1371/journal.pgen.1005680)
Supplement: S5 Table — (DOCX) [file pgen.1005680.s011.docx]

**S5 Table. Sequences associated with murine H3K27ac peaks proximal to the promoter regions of *Skap*2 and *Creb5***

| Skap2 Promoter H3K27ac peak 1  >mm9_dna range=chr6:51962758-51962932 5'pad=0 3'pad=0 strand=- repeatMasking=none  CCAGACGGGGGTTTAAACCTTGGTGGAAAAGACCAGTATTTAGATGACCCCTTAAAGGGAAAGTGCGTAAGCCTTGCGGAGGCTTTATTTTCCGGAATCCTGGAGAAAGAGAGGTGTGGCTGACATACCAGTAACGAAAATGGCGCGGACGAGCTTCAAGGGCCGGTCAGGTGAC  Creb5 Promoter H3K27ac peak 1  >mm9_dna range=chr6:53523083-53523242 5'pad=0 3'pad=0 strand=- repeatMasking=none  CCTCTGGCTCTGCTGCTCACTCTCTTTCTCCTGGGCTCGCCCTCCTCCCC  CTTCTCCTTTTTAGCTCAGCGCTGGTGAAGTCACTATTTAAATCTGGCAG  AACTGAAGCAAAAACTTCAATGTAACCAAAACAGCCCCAGGCCCAGTTCCAGGCTCAGGG  Creb5 Promoter H3K27ac peak 2  >mm9_dna range=chr6:53522918-53523064 5'pad=0 3'pad=0 strand=- repeatMasking=none  CCTTAGAAACCAGTAAACAGACTAAGAAACAGTGCAGCCTGGGGATGGCCTACAGCTGCCATTGGAGCTCAGAGTGGGACCCCCGGTGTCTGTGACCAGGGGTGGCATTGTTCTTCTAGAAACAAATA |
| --- |
|  |
